# Atomistic to Mesoscopic Modelling of Thermophysical Properties of Graphene-Reinforced Epoxy Nanocomposites

**DOI:** 10.3390/nano13131960

**Published:** 2023-06-28

**Authors:** Atta Muhammad, Carlos Sáenz Ezquerro, Rajat Srivastava, Pietro Asinari, Manuel Laspalas, Agustín Chiminelli, Matteo Fasano

**Affiliations:** 1 Department of Energy, Politecnico di Torino, 10129 Torino, Italy; atta.muhammad@polito.it (A.M.); rajat.srivastava@unisalento.it (R.S.); pietro.asinari@polito.it (P.A.); 2 Department of Mechanical Engineering, MUET SZAB Campus, Khairpur Mir’s 66020, Pakistan; 3 Aragon Institute of Technology ITAINNOVA, 50018 Zaragoza, Spain; csaenz@itainnova.es (C.S.E.); mlaspalas@itainnova.es (M.L.); achiminelli@itainnova.es (A.C.); 4 Department of Engineering for Innovation, University of Salento, 73100 Lecce, Italy; 5 Istituto Nazionale di Ricerca Metrologica, 10135 Torino, Italy

**Keywords:** polymer, thermosets, graphene, nanocomposite, coarse-grained molecular dynamics, continuum simulation, thermal conductivity, elastic matrix

## Abstract

This research addresses the need for a multiscale model for the determination of the thermophysical properties of nanofiller-enhanced thermoset polymer composites. Specifically, we analyzed the thermophysical properties of an epoxy resin containing bisphenol-A diglyceryl ether (DGEBA) as an epoxy monomer and dicyandiamide (DICY) and diethylene triamine (DETA) as cross-linking agents. The cross-linking process occurs at the atomistic scale through the formation of bonds among the reactive particles within the epoxy and hardener molecules. To derive the interatomic coarse-grained potential for the mesoscopic model and match the density of the material studied through atomic simulations, we employed the iterative Boltzmann inversion method. The newly developed coarse-grained molecular dynamics model effectively reproduces various thermophysical properties of the DGEBA-DICY-DETA resin system. Furthermore, we simulated nanocomposites made of the considered epoxy additivated with graphene nanofillers at the mesoscopic level and verified them against continuum approaches. Our results demonstrate that a moderate amount of nanofillers (up to 2 wt.%) increases the elastic modulus and thermal conductivity of the epoxy resin while decreasing the Poisson’s ratio. For the first time, we present a coarse-grained model of DGEBA-DICY-DETA/graphene materials, which can facilitate the design and development of composites with tunable thermophysical properties for a potentially wide range of applications, e.g., automotive, aerospace, biomedical, or energy ones.

## 1. Introduction

In recent years, there has been a growing interest among researchers and industrialists in polymer nanocomposites, which are composed of polymers that incorporate nanofillers and have superior mechanical, electrical, and thermal properties compared to neat polymers. Various types of nanofillers and additives, such as carbon nanofibers, graphene, graphene oxide, iron, iron oxide, silica, and alumina nanoparticles, are utilized in conjunction with thermoset polymers, such as epoxies, as matrix materials to form nanocomposites [1,2,3]. Carbon nanofillers, with their distinctive physical characteristics, are preferred for creating high-performance epoxy nanocomposites compared to other nanofillers [4,5,6]. These nanocomposites have garnered significant attention from both academic and industrial sectors due to their broad range of potential uses [7,8,9]. Furthermore, graphene and graphene oxide have emerged as promising candidates for a wide range of applications, such as communication, photodetectors, and chemical sensors [10,11]. These materials also hold promise in space exploration and biotechnology [12].

Epoxy resins are produced through cross-linking between epoxy monomers with multiple epoxide groups and hardeners containing active groups such as hydroxyls and amines. The stable covalent bonds among the different chains in cured epoxy resins often result in superior thermophysical characteristics [1]. Therefore, they are being utilized in various industries, such as electronics, automotive, and aerospace, due to their good mechanical strength and the opportunities that give in terms of weight reduction when used in composites [13,14].

The thermophysical properties of nanocomposites play a critical role in designing high-performance smart applications and verifying the integrity of structures or devices. Baptista et al. [15] investigated the mechanical properties of graphite-reinforced epoxy nanocomposites and found that the elastic modulus increases with an increase in graphite content. Zhao et al. [16] and Ma et al. [17] synthesized amino-functionalized CNT (a-CNT) reinforced epoxy composites. They reported an enhancement in the ultimate tensile strength, elastic modulus, and storage modulus by 25%, 30%, and 10%, respectively, when 0.5 wt% a-CNT was added to the epoxy. Wang et al. [18] developed epoxy/graphene nanosheet (GNS) composites to study their effective electromagnetic shielding properties over the 8–20 GHz frequency range. The minimum reflection loss of the epoxy/GNS composite reached −14.5 dB at a frequency of 18.9 GHz when 15 wt% GNS was added. This can be attributed to dielectric loss caused by enhanced electric conductivity and charge multipoles at the polarized contacts. Similarly, Wang et al. [19] evaluated the thermal properties of epoxy/GNS composites. The thermal conductivity of epoxy/GNS composites increased significantly with an increase in GNS loading. Reference [19] reported a 115% increase in thermal conductivity when 5 wt% GNS was added to neat epoxy.

Experimental procedures are often costly, and therefore, physics-based computer modeling has been developed as a valuable complement to experimental and analytical methods. In recent years, molecular dynamics (MD) simulations have emerged as a promising tool for predicting material properties using well-parametrized and validated interatomic potentials. MD simulations have also been employed to investigate thermoset polymers. Fully atomistic models have been developed through MD simulations to evaluate various characteristics of epoxies [20,21,22,23].

Gavrielides et al. [20] investigated the glass-transition temperature and coefficient of thermal expansion of the DGEBA-EDA epoxy resin model using classical molecular dynamics simulations and a generalized AMBER force field [24] to describe inter- and intramolecular interactions of the epoxy resin. The resulting densities, glass-transition temperature, and coefficient of thermal expansion were in good agreement with the literature. Bandyopadhyay et al. [21] created an epoxy resin based on diglycidyl ether of bisphenol-F (EPON 862) and the hardener diethylene toluene diamine (DETDA) using atomistic modeling. They employed an OPLS force field [25] and determined the glass-transition temperature, thermal coefficient of expansion, and elastic properties of the cross-linked systems. The study found that the glass-transition temperature and elastic stiffness tended to increase with the cross-linking degree, while the coefficient of thermal expansion above and below the glass-transition temperature decreased. Li et al. [22] employed a DREIDING force field [26] to simulate the EPON 862-DEDTA epoxy resin system and investigate the influence of curing on thermomechanical properties. The model indicated that the glass-transition temperature, stiffness, and yield stress strongly depend on the degree of polymerization. Nejad et al. [23] conducted atomistic simulations to evaluate the thermal characteristics of DGEBA-DETA epoxy resin reinforced with carbon nanotubes. To gain a comprehensive understanding of how thermal conductivity originates from composite characteristics at the nanoscale, the thermal properties of the components were independently studied with respect to various chemical, geometrical, and physical features. MD simulations were first employed to investigate the thermal conductivity of epoxy resin and carbon nanotubes. Subsequently, the Kapitza resistance at the interfaces of epoxy resin and carbon nanotubes was examined. Finally, the thermal conductivity of the epoxy/CNT nanocomposites was calculated, and the observed behavior was evaluated based solely on the matrix, nanofillers, and interface characteristics. The results showed that the thermal conductivity of the nanocomposite increases with the volume fraction, carbon nanotube length, system temperature, and curing degree, as also predicted by the effective medium theory.

Although MD simulations have been widely used to predict and interpret various heat and mass transfer mechanisms at the nanoscale, full atomistic models are subject to significant time and scale constraints, making them unsuitable for accurately modeling the thermal and mechanical characterization of polymers and nanocomposites with elevated concentrations of fillers. Coarse-graining (CG) is a technique for upscaling an atomistic model by grouping multiple atoms into a single entity, resulting in reduced degrees of freedom and larger timesteps with respect to MD simulations owing to the softness of interatomic potentials. The CG approach comprises two primary steps: (i) grouping the model into larger elements rather than single atoms, and (ii) establishing interparticle potentials, which are highly dependent on the material and application. The literature describes a set of procedures that classify the iterative determination of effective pair potentials for particles by accurately matching the material’s structural properties, such as its radial distribution function (RDF), beginning with the potential of mean forces derived via Monte Carlo (MC) or Boltzmann inversion techniques [27,28,29,30]. Precisely evaluating both thermodynamic and structural characteristics in coarse-grained (CG) models is a challenging task. Poor conformity with one property can often result in excellent agreement with the other property. Consequently, determining the optimal CG approach relies on which properties are of the greatest interest. A popular coarse-grained force field is the MARTINI one [31,32]. Specifically designed for surfactant and lipid systems, the MARTINI force field parameters are determined based on the free energy partitioning between the polar and apolar phases of various chemical substances. In this model, each interaction center represents four heavy atoms and their associated hydrogens.

Regarding previous CG simulations of thermoset composites, Komarov et al. [33] employed a reverse mapping strategy to bridge atomistic and mesoscopic models of epoxy resin systems. They used MC simulation to cross-link epoxy monomers. The developed mesoscopic model predicted the increase in the glass-transition temperature with the degree of cross-linking in agreement with experimental data. However, the volume expansion coefficient was approximately 30% higher than the experimental value. Yang et al. [14] designed a mesoscopic model for epoxy resin comprising phenol novolac (EPN) as the epoxy monomer and bisphenol-A (BPA) as the curing agent. They parameterized the potentials by fitting them to important thermomechanical characteristics such as glass-transition temperature, elastic constant, and Poisson’s ratio, determined through full atomistic simulations and experiments. The developed CG model of epoxy resin could predict various thermomechanical characteristics. Later, Fu et al. [13] conducted non-equilibrium CG molecular dynamics simulations under varying degrees of cross-linking to investigate the rheological behavior of the EPN-BPA epoxy. They used potential parameters different from those initially proposed by Yang et al. [14].

**Figure 1:**
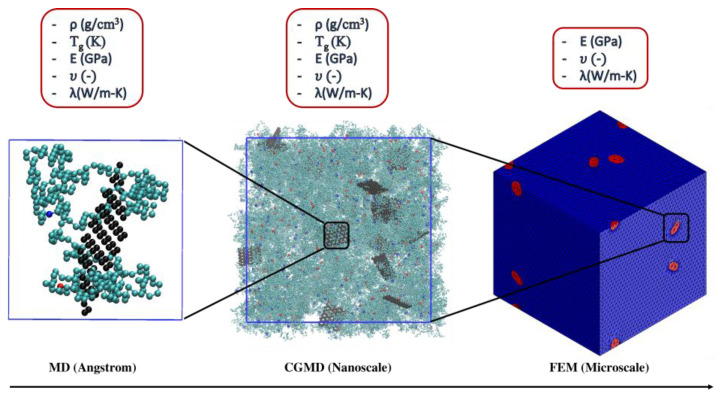
Schematic diagram of modeling a nanocomposite across different scales.

Hence, provided that appropriate material modeling tools are available, understanding and predicting the properties of graphene-reinforced epoxy nanocomposites across different lengths and time scales can potentially facilitate the development of innovative materials. As depicted in Figure 1, the current study develops an atomistic-to-continuum bridging approach to the thermophysical properties of an epoxy resin containing bisphenol-A diglyceryl ether (DGEBA) as an epoxy monomer and dicyandiamide (DICY) and diethylene triamine (DETA) as cross-linking agents. To this purpose, a novel mesoscopic model of the considered epoxy resin was developed based on atomistic simulations. To validate the results of the CG MD simulations, comparisons were made with full atomistic MD simulations as well as existing mesoscopic and experimental studies. The interparticle interactions obtained by the mesoscopic model were utilized to model the influence of graphene filler on the thermophysical behavior of the epoxy resin at the mesoscopic level. Additionally, the mesoscopic results were used as inputs for the continuum-scale model [34,35] to complement and verify the results obtained at this coarser level.

## 2. Materials and Methods

### 2.1. Atomistic Model of Epoxy

**Figure 2:**
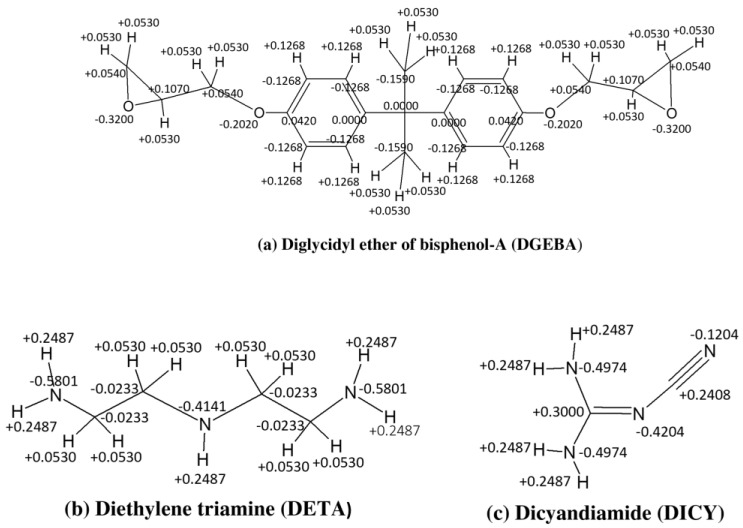
Chemical structures and partial charges (in eV) for (**a**) Diglycidyl ether of bisphenol-A (DGEBA), (**b**) Diethylene triamine (DETA), and (**c**) Dicyandiamide (DICY).

The epoxy system investigated in this study is made of DGEBA as the epoxy monomer and DICY and DETA as the curing agents. The DGEBA-based epoxy system is a preferred choice for high-performance applications due to its superior mechanical and thermal properties, excellent adhesion to various substrates, and chemical resistance [36,37,38]. Its low viscosity enables easy processing and improved mechanical properties of composite materials [39]. Focusing on this epoxy system can lead to innovations in the development of new materials and improvements in existing products. Figure 2 provides the chemical structures and partial charges of these compounds. For atomistic molecular dynamics simulations, the partial charges were computed using the bond increments specified in the COMPASS force field for condensed-phase optimized molecular potentials [40]. The curing process takes place between the nitrogen atoms (N) in the amino groups of DICY and DETA and the electrophile carbon atoms (C) in the epoxy groups of DGEBA. The cut-off distance ($R_{cut}$) is a crucial factor that determines the success of the curing reaction, as atoms with a distance of less than $R_{cut}$ are linked together. The atom charges are modified to accommodate the new functional groups and bond configurations resulting from the curing process (refer to Supplementary Note S1 for further details).

### 2.2. Mesoscopic Model of Epoxy

**Figure 3:**
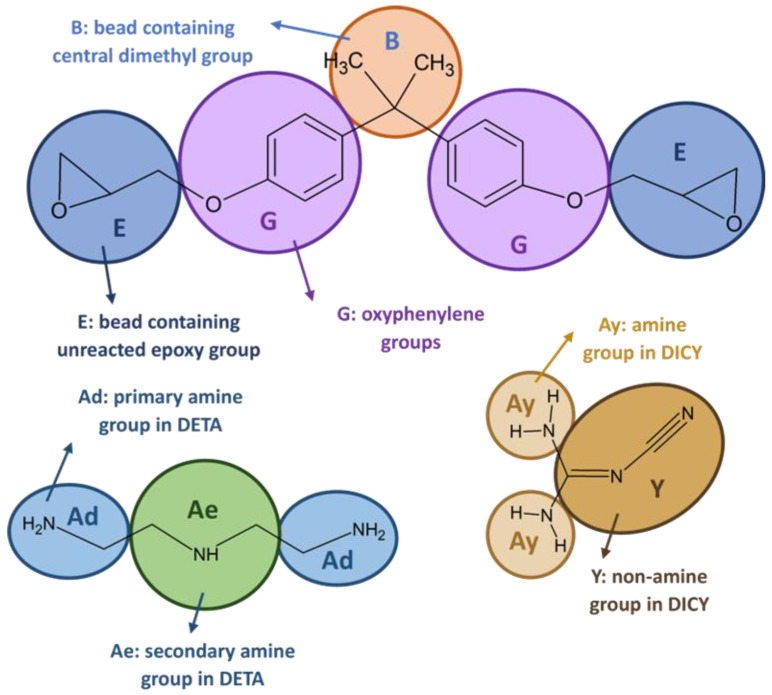
Mapping scheme for epoxy resin and hardener molecules.

The mesoscopic model is characterized by the atomic composition of the CG beads, their interconnections, as well as bonded and non-bonded potential interactions. The initial stage involves atoms-to-bead mapping, wherein atoms are categorized into respective beads. In the case of the DGEBA model, atoms comprising the epoxy group were assigned to E beads, oxyphenylene groups to G beads, and dimethyl groups to B beads. In the DETA model, primary and secondary amine groups were assigned to Ad and Ae beads, respectively. Finally, in the DICY model, amine groups were assigned to Ay beads, whereas non-amine groups were assigned to Y beads (see Figure 3).

During the curing process, reactive beads of DGEBA, DETA, and DICY form bonds, where beads Ad, Ae, and Ay (based on amines) bond with E beads, leading to the formation of a cross-linking point. Beads Ad and Ay have the potential to react twice, resulting in the formation of Ad1, Ad2, Ay1, and Ay2 beads (refer to Supplementary Note S1 for further details). The cross-linking degree is determined by dividing the number of reacted E beads (E′) by the total number of E beads.

### 2.3. Coarse-Graining by Iterative Boltzmann Inversion

A coarse-graining technique was utilized to extract the mesoscopic force field of the epoxy resin from the representative full-atom model. The potential parametrization process comprised several stages, which are elaborated below and began with the Iterative Boltzmann Inversion (IBI) method. The IBI method is a structure-based coarse-graining method that determines the effective pair potential of particles in thermal equilibrium. It efficiently calculates potentials for the coarse-grained model based on structural characteristics, such as the radial distribution function (RDF), which is derived from atomistic simulations [41]. According to the Henderson theorem [42], the coarse-grained potential is unique, which means that there is only one pair interaction potential that can yield the desired RDF for any given RDF.

After generating the RDF for a specific degree of freedom (such as bond length, angle, or distance between non-bonded beads) from the atomistic configuration, Boltzmann inversion is applied to obtain the corresponding interaction potential, that is:

(1) $$U\left(q\right)=k_{B}T\ln P\left(q\right),$$

where the potential energy ($U\left(q\right)$) is defined in terms of the degree of freedom q, the Boltzmann constant ($k_{B}$), the system temperature (T), and the probability of the degree of freedom ($P\left(q\right)$). Direct Boltzmann inversion is sufficient for bonded interactions such as bonds and angles, as the corresponding RDF is usually symmetric or near-Gaussian [43]. However, for non-bonded interactions such as Coulombic and van der Waals, direct inversion does not accurately reproduce a potential curve corresponding to the target RDF obtained from atomistic simulation. In these cases, an iterative refinement process can be employed to introduce a corrective term to the interaction potential [43], namely:

(2) $$U^{\left(i+1\right)}=U^{\left(i\right)}+\lambda k_{B}T\ln\frac{g_{i}^{CG}\left(r\right)}{g_{*}^{CG}\left(r\right)\;}.$$

The iterative process involves refining the CG potentials obtained from direct inversion by applying a correction term. The correction is gradually reduced as the $i^{\mathrm{th}}$ iteration of the radial distribution function $g_{i}^{CG}\left(r\right)$ approaches the target one $g_{*}^{CG}\left(r\right)$ corresponding to that computed by MD simulations. The stabilization factor λ is applied to avoid undesired instabilities. The factor λ is initially set to 0.45 and increased as $g_{i}^{CG}\left(r\right)$ approaches $g_{*}^{CG}\left(r\right)$. The potentials are updated independently for each specific interaction.

The iterative Boltzmann inversion method yields potentials that may not accurately reproduce the thermodynamic behavior of the system, such as the system pressure. To overcome this limitation, an additional correction term can be introduced as follows:

(3) $$\Delta U\left(r\right)=-f\frac{P_{i}-P_{0}}{P_{0}}k_{B}T\left(1-\frac{r}{r_{\mathrm{cutoff}}}\right),$$

where f is a stabilization factor (set to $1\times 10^{-5}$), $P_{i}$ is the pressure of the *i*th iteration of potential refinement, $P_{0}$ is the target pressure (usually 1 atm), r represents the distance between two beads, and $r_{\mathrm{cutoff}}$ is the cut-off distance (set at 18.05 Å in this study). Note that this correction term becomes negligible at $r_{\mathrm{cutoff}}$.

**Figure 4:**
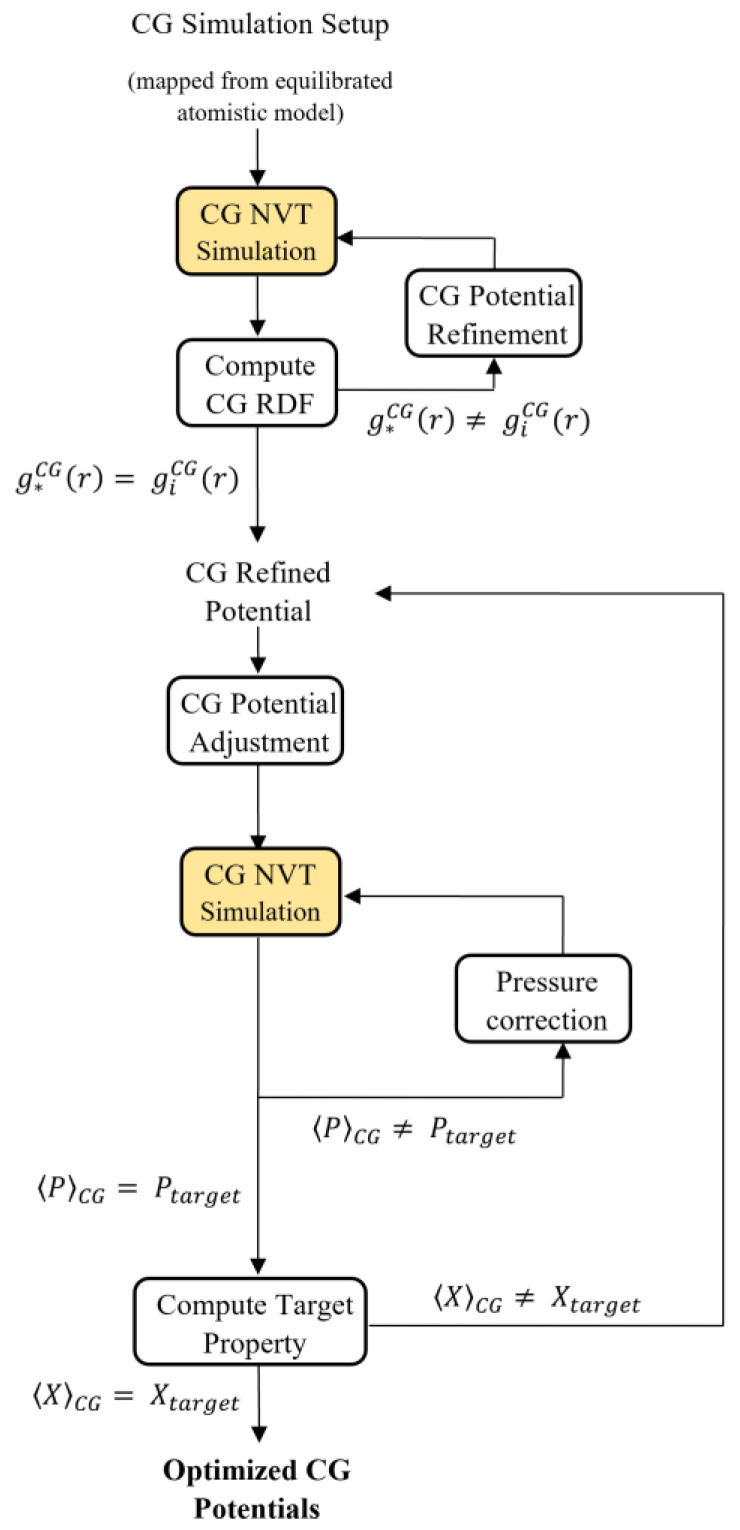
The coarse-graining procedure based on the Iterative Boltzmann Inversion method.

After adjusting the pressure of the coarse-grained system, the target property ($X_{\mathrm{target}}$), which could be a mechanical or thermal property, was evaluated by prolonged simulation. Figure 4 illustrates the overall coarse-graining process.

### 2.4. Mesoscopic Model of Graphene

**Figure 5:**
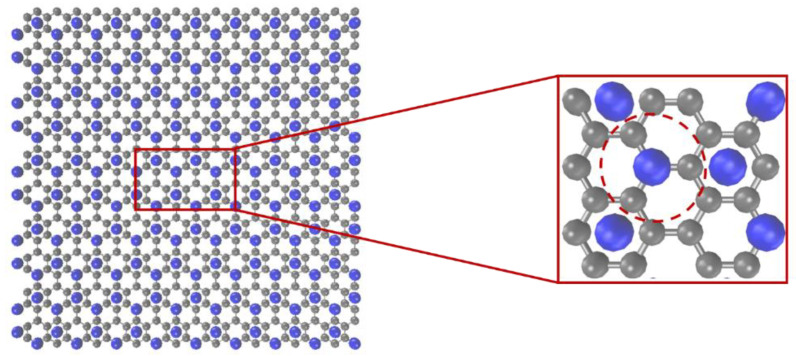
Mesoscopic model of graphene (blue beads) from atomistic details (gray atoms).

Graphene (Gr) is a material known for its exceptional mechanical strength and high surface-to-weight ratio, making it an excellent filler for nanocomposites [44]. Despite significant progress in synthesizing large-scale graphene sheets and assemblies, their macroscale properties depend heavily on the size and arrangement of the graphene. Therefore, understanding the relationship between microstructural evolution and macroscopic behavior is crucial. However, experimental techniques may have limited resolution while studying molecular processes [45]. While full-atomic MD simulations provide detailed information [46,47], they can be time-consuming. To overcome this, a computationally efficient technique such as the CG MD method has been proposed. Here, a CG force field was developed for neat graphene based on the TersoffCG potential [48]. In this force field, each CG bead represents four carbon atoms from the full-atomistic model, as depicted in Figure 5. Consequently, the bead mass is four times that of a single carbon atom, and the spacing between neighboring beads is double (0.284 nm) the original atomic distance (0.142 nm). This mesoscopic model retains the same structure as the atomistic model of graphene but has three-quarters of the degrees of freedom.

The TersoffCG potential is directly derived from the full-atomistic Tersoff potentials [49] by adjusting some force field parameters (see Supplementary Note S2 for a detailed discussion). Given that in the TersoffCG potential one bead represents 4 carbon atoms of the full-atom model, then the parameters A and B in the TersoffCG potential were obtained by multiplying four times the values in the Tersoff potential. The $\lambda_{1}$, $\lambda_{2}$, and $\lambda_{3}$ values are half of the Tersoff potential since they have measured units of ${Å}^{-1}$. However, given that the geometry of the CG model remains the same as in the full-atom model, the parameters related to bonds and angles did not require adjustment, and the γ, c, d, and $\cos\theta_{0}$ parameters remained constant. To recreate some of the properties of pristine graphene, the $R\left(Å\right)$ and $D\left(Å\right)$ parameters had to be tuned. The stress-strain plot of the atomic model served as a benchmark for optimization. The values of R and D were varied to determine their impact on the CG model stress-strain curve, and the best value was selected. The TersoffCG potential was found to provide the best fit to the stress-strain curve with R=3.9 Å, and D=0.3 Å. A summary of the parameter values used in the CG potential obtained is presented in Supplementary Note S2.

### 2.5. Simulation Protocol for Atomistic and Mesoscopic Simulations

**Figure 6:**
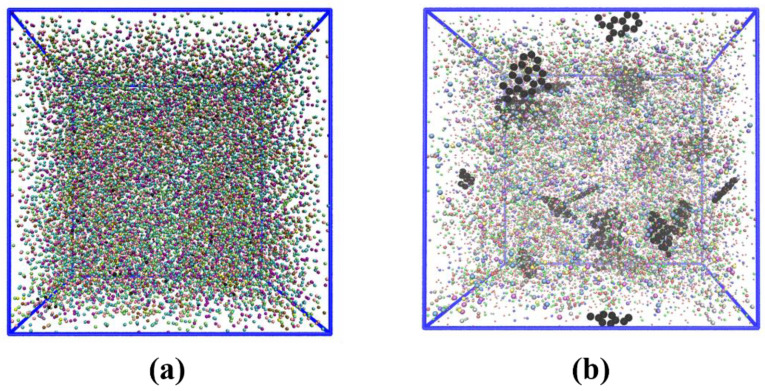
Equilibrated cross-linked CG model for (**a**) epoxy resin [113 × 113 × 113 Å^3^] and (**b**) epoxy/Gr nanocomposite (2.0 wt.% Gr) [115 × 115 × 115 Å^3^].

In the network structure of epoxy-based materials, the curing reaction between epoxy and hardener monomers is a crucial step that should be accurately reflected in molecular dynamics simulations, whether atomistic or coarse-grained. Cross-linking was performed separately for the atomistic and CG models to ensure consistency and reproducibility. To achieve this, a reactant mixture containing stoichiometric amounts of DGEBA, DICY, and DETA (100:5:5) was randomly seeded into a cubic box at low density using LAMMPS (large-scale atomic molecular massively parallel simulator) [50]. A multistep cross-linking process was then performed using a LAMMPS script, based on the chemical reactivity of amine groups towards epoxydic groups, to form a completely cross-linked system. In this process, pairs of unreacted atoms within a search radius are detected, and their identities are modified to cross-link them. The cross-linking procedure in the CG model involves bonding between reactive CG beads and bead-type reassignment resulting from the formation of the cross-linked bonds. The cut-off distance between reacting particles is set at 6.0 Å. Upon reaching the target cross-linking degree of 70%, updates to the new angles and bead charges were made on their respective types. To obtain a completely cross-linked system with minimized energy, some equilibration runs were subsequently conducted utilizing both NVT and NPT ensembles, under the control of a Nosé-Hoover thermostat and barostat, while maintaining the system at a temperature of 300 K and a pressure of 1 atm. The resulting equilibrated systems, obtained through both atomistic (53 × 53 × 53 Å^3^) and CG simulations (113 × 113 × 113 Å^3^), as depicted in Figure 6, were utilized for the determination of the material’s thermophysical properties. The process described here is consistent with the works of Jang et al. [51], Sirk et al. [52], and Gavrielides et al. [20].

Following the construction of the cross-linked epoxy resin system for both atomistic and coarse-grained models, the density was measured at different temperatures, ranging from 350 K to 430 K, considering the NPT ensemble. To ensure equilibrium, the system underwent a 5 ns equilibration process in each instance. The glass-transition temperature was then determined by analyzing the discontinuity of the density as a function of temperature.

Subsequently, the elastic (Young) modulus and Poisson’s ratio of the epoxy resin were computed using molecular dynamics simulations, where the system was subjected to uniaxial deformation and the mechanical response was recorded. The Young modulus and Poisson’s ratio were obtained from the uniaxial tensile deformation simulations conducted in all three directions (*x*, *y*, and *z*) to obtain an average value along with standard deviation for the computed properties. This deformation process follows Hooke’s law:

(4) σ=Eϵ,

where σ represents the stress vector, ϵ is the strain vector, and E the stiffness matrix. As epoxies are considered isotropic materials, their properties remain unchanged in all directions. This means that they have only two independent elastic constants (Young’s modulus E and Poisson’s ratio ν) in their stiffness and compliance matrices.

To investigate the thermal conductivity of the system, we employed a Müller–Plathe method based on non-equilibrium molecular dynamics (NEMD) simulations [53] in all three directions (*x*, *y*, and *z*) to obtain an average value along with standard deviation. The method involved creating two lower-temperature regions at opposite ends of the simulation box and imposing a certain amount of heat in the middle region (i.e., the higher-temperature section). As a result, a temperature gradient was established in the model, and heat flow occurred throughout the simulation domain via velocity exchanges between the atoms of the “cold” and “hot” regions. Periodic boundary conditions were applied in all directions (i.e., *x*, *y*, and *z*). Once the system reached a steady state, the amount of energy transferred per unit of time and cross-sectional area from the hot region to the cold region was calculated as:

(5) $$j_{z}=\frac{1}{2tA}\underset{\mathrm{transfers}}{\sum }\frac{m}{2}\left(v_{\mathrm{hot}}^{2}-v_{\mathrm{cold}}^{2}\right),$$

where t represents the simulation time, A the cross-sectional area normal to the heat flux direction, m mass, $v_{\mathrm{hot}}$ and $v_{\mathrm{cold}}$ are the velocities of the atoms in the hot and cold regions, respectively. The factor of 2 is due to the energy flow occurring in two directions from the hot to the cold region. The thermal conductivity (λ) of the system can be determined from the heat flux ($j_{z}$) and temperature gradient (∂T/∂z) using Fourier’s law given as [53]:

(6) $$\lambda =\underset{t\to \infty }{lim}-\frac{\langle j_{z}(t)\rangle }{\langle \partial T/\partial z\rangle }.$$

In addition to thermal conductivity, this study also aimed to determine the specific heat capacity of the model at constant pressure. To achieve this, we recorded the average enthalpy and temperature at different temperatures ranging from 300 K to 350 K, considering the NPT ensemble. The resulting data were then plotted as an enthalpy-temperature plot. The slope of this plot provided the specific heat capacity $\left(C_{p}\right)$ of the material.

### 2.6. Continuum Models of Epoxy/Gr Composites

**Figure 7:**
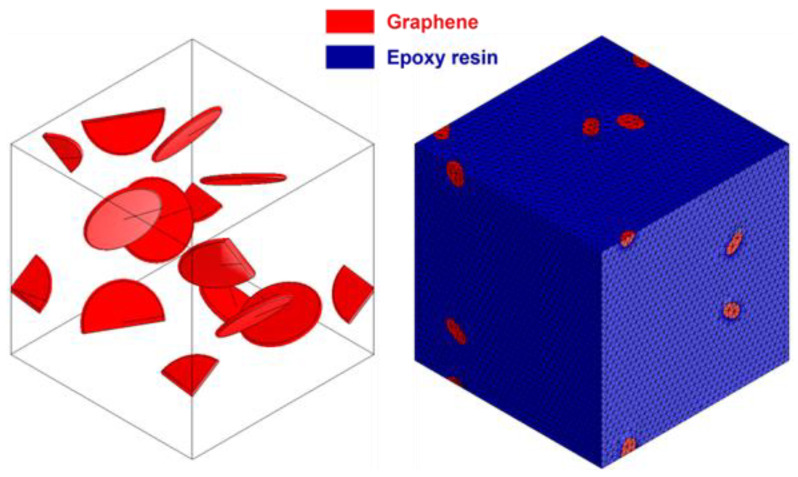
RVE continuum model of epoxy/Gr composite.

The predicted thermophysical properties of epoxy/Gr nanocomposites obtained from the developed mesoscopic model were then compared with two continuum models, namely mean field (MF) and finite element method (FEM). The MF homogenization approach utilized first-order Mori and Tanaka [54] (based on an approximation of the Eshelby solution [55]). Representative volume element (RVE) models were generated for the epoxy/Gr nanocomposites to carry out finite element analysis. The constitutive relationship of the RVE model with isotropically symmetric elements within nanocomposites was established using the generalized Hook’s law. The elastic modulus, Poisson’s ratio, and thermal conductivity of the models were calculated. The RVE model employed in this study was made up of a DGEBA-DETA-DICY epoxy matrix and graphene inclusions (platelet-shaped) with a completely bonded interface and an aspect ratio of 10 (such as the CG model). A tetra-conforming mesh with quadratic elements, internal coarsening, and curvature control was used to randomize the distribution of the inclusion phase by assigning the same mass fraction of CG MD simulation. Figure 7 depicts the RVE model used in this study, which includes periodic boundary conditions.

## 3. Results and Discussion

### 3.1. Atomistic Simulation of Epoxy

**Figure 8:**
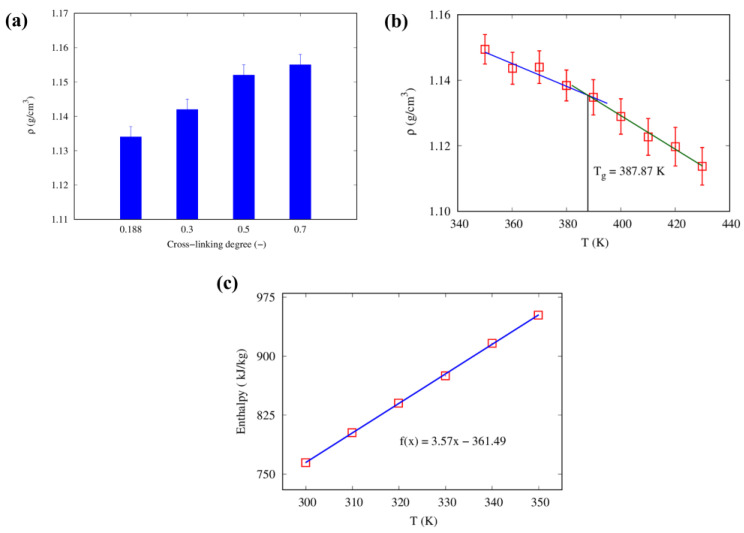
(**a**) Density as a function of the degree of cross-linking, (**b**) density as a function of temperature, and (**c**) enthalpy-temperature plot for the atomistic model of epoxy resin, with best fitting function.

We constructed four epoxy models with varying degrees of cross-linking (20%, 30%, 50%, and 70%). To prepare these models, we first randomly distributed the constituent monomers of the epoxy resin in stoichiometric proportion (calculated as 100:5:5 of DGEBA:DICY:DETA by weight) within a simulation box. The time integration was applied first in the NVT ensemble and later in the NPT ensemble at a temperature of 300 K and pressure of 1 atm. After this initial distribution and equilibration, we applied a cross-linking procedure to achieve the desired cross-linking degree of the epoxy resin. The model consisted of approximately 13,500 atoms. We used the density of the epoxy resin model as an indicative parameter to assess its validity. The densities of the epoxy models with 20%, 30%, 50%, and 70% cross-linking degrees were found to be 1.134 g/cm^3^, 1.142 g/cm^3^, 1.152 g/cm^3,^ and 1.155 g/cm^3^, respectively. As depicted in Figure 8a, the density increased as the degree of cross-linking increased, given the higher amount of bonds between the resins and the hardeners. Our proposed model agrees well with published atomistic and experimental epoxy densities, which range from 1.07 to 1.20 g/cm^3^ [56,57,58].

To determine the glass-transition temperature of the epoxy resin model with a 70% cross-linking degree, we conducted equilibrium simulations considering the NPT ensemble. Specifically, we progressively increased the system temperature from 350 K to 430 K in steps of 10 K over a period of 2 ns while maintaining a pressure of 1 atm. From these simulations, we obtained densities at various temperatures and plotted the resulting density-temperature curve. By identifying the junction point of the two fitting lines in the density-temperature plot, we calculated the glass-transition temperature. As shown in Figure 8b, the glass-transition temperature for the 70% cross-linked system was found to be 387 K. This value agrees well with published atomistic results as well as experimental results (380–390 K) [20].

The mechanical properties of the epoxy resin were determined from stress-strain curves. The elastic modulus of the 70% cross-linked epoxy resin was found to be 2.99 GPa using the linear theory of elasticity, which agrees well with the range of experimental values from 2.5–5.0 GPa [56,57,58,59,60,61,62]. Poisson’s ratio was also calculated and found to be 0.293, consistently with the experimental range of 0.22–0.33 [57,59,61].

Additionally, atomistic simulation was performed to calculate the thermal conductivity of the 70% cross-linked epoxy model, resulting in an average thermal conductivity of 0.163 W/mK at 300 K, which is in close agreement with literature values ranging from 0.18–0.2 W/mK [63,64]. The specific heat capacity at a constant pressure of the equilibrated model was evaluated from the enthalpy-temperature plot, yielding a slope of 3.57 kJ/kgK from the linear fit of the data in Figure 8c, which is higher than the experimental range of 1.0–2.0 kJ/kgK [65,66]. This discrepancy may be due to the omission of quantum effects in the MD simulations used to calculate the specific heat capacity, resulting in an overestimation of its value—coherently with previous observations in the literature [67,68].

### 3.2. Coarse-Grained Potentials Determination

**Figure 9:**
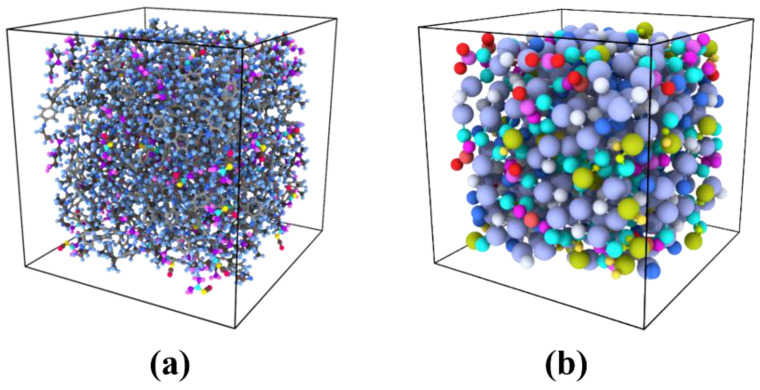
(**a**) Atomistic and (**b**) coarse-grained (obtained from mapping) model of epoxy resin used for the coarse-graining procedure (cross-linking degree equal to 0.5).

To develop the coarse-grained force field for the epoxy resin, an atomistic model was specifically designed with a degree of cross-linking of 50%, as illustrated in Figure 9. In the model, non-periodic boundaries condition has been applied to allow for precise monitoring of the positions of atoms (and corresponding beads) and bonds, thereby obtaining an accurate radial distribution function (RDF).

Supplementary Note S3 provides a comprehensive set of potentials that were developed for the coarse-grained epoxy resin. Bond, angle, and non-bonded potentials were established with the distinct beads defined in the model. The non-bonded potentials were truncated at a cut-off distance of 18.05 Å, beyond which no interaction between beads occurred. The bond and angle potentials were directly derived using the Boltzmann inversion and did not require refinement through the iterative IBI method. Conversely, the iterative IBI method was utilized to develop the non-bonded potentials because direct inversion alone was inadequate for achieving a good match with the target RDF. To simplify the potential development, the reacted bead types (E′, Ad1, Ad2, Ae1, Ay1, and Ay2) were treated as equivalent to their non-reacted counterparts (E, Ad, Ae, and Ay), which reduced the number of potentials required. The IBI method yielded 28 non-bonded potentials, and the pressure correction procedure was applied once the appropriate degree of fitting with the target RDF function was achieved.

Together with the coarse-graining of potentials, we developed a specific cross-linking procedure based on LAMMPS scripts for the epoxy resin CG force field. Following cross-linking, the new angle types generated through the procedure were assigned, and the charges of beads were updated according to their types. This method enables more accurate and efficient simulation of cross-linked epoxy systems using the developed CG force field. To assess the stability of the CG force field, a long simulation was conducted on a cross-linked CG epoxy system using a time step of 1.0 femtoseconds (twice the value used in full atomistic simulations). The simulation results, presented in Supplementary Notes S3 and S4, demonstrated stable density after 0.1 ns, and the system remained stable for at least 1.2 ns.

### 3.3. Mesoscopic Simulation of Epoxy

**Figure 10:**
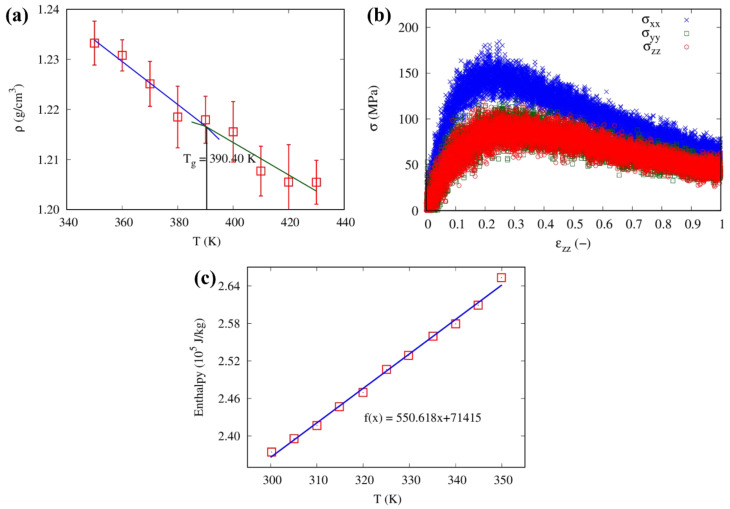
(**a**) Density as a function of temperature, (**b**) stress vs. strain curve, and (**c**) enthalpy–temperature plot for the CG model of epoxy resin, with best fitting functions.

The accuracy of the CG model was assessed by comparing the density, glass-transition temperature, elastic modulus, Poisson’s ratio, thermal conductivity, and specific heat capacity against both atomistic simulation and experimental values from the literature. This investigation focused on the CG epoxy model with 70% cross-linking, as Fourier-transform infrared spectroscopy (FTIR) studies monitoring the curing process of epoxy resins have indicated that the cross-linking degree typically falls between 50% to 100%, depending on the temperature [69]. After cross-linking the model, the density was determined to be 1.24 g/cm^3^ at 300 K and 1 atm, which is in good accordance with atomistic simulation and experimental values ranging from 1.07 to 1.20 g/cm^3^ [56,57,58]. The equilibration procedure was then carried out to determine the glass-transition temperature. The temperature was increased from 350 K to 430 K with a step of 10 K for 2 ns. The glass-transition temperature was calculated using densities at various temperatures obtained from an NPT ensemble at a pressure of 1 atm. The junction point of the two fitting lines in the density-temperature plot, as shown in Figure 10a, was used to calculate the glass-transition temperature for such a 70% cross-linked system. The obtained value (390 K) is in good agreement with full atomistic and experimental results (380–390 K) [20].

The Young’s modulus and Poisson’s ratio were obtained from the stress-strain plot using the CG model developed here, as shown in Figure 10b. Stress-strain plots were obtained from the uniaxial tensile deformation. The elastic modulus of the CG epoxy with 70% cross-linking degree was found to be 1.419 GPa, which is lower than the atomistic simulation (2.9 GPa) and literature values (2.5–5.0 GPa) [56,57,58,59,60,61,62]. The Poisson’s ratio was around 0.33, which aligns well with the atomistic simulation (0.29) and experimental values (0.22–0.33) [57,59,61]. The average value of thermal conductivity at 300 K was found to be 0.10 W/mK, which is lower than atomistic (0.16 W/mK) and literature values (0.18–0.2 W/mK) [63,64]. The specific heat capacity at a constant pressure of the equilibrated model was evaluated from the slope of the enthalpy-temperature curve, see Figure 10c for the 70% cross-linked CG epoxy model. Linear fitting of this enthalpy-temperature curve resulted in a slope of 0.55 kJ/kgK. The reduction of degrees of freedom at the CG level accounts for the observed discrepancy between mechanical and thermal properties obtained at atomistic and CG levels, as similarly noticed in previous works [68,70].

A tabulated comparison between the atomistic and mesoscopic model predictions, as well as literature measures for the thermophysical properties of the considered epoxy, are reported in Supplementary Note S5.

### 3.4. Mesoscopic Simulation of Graphene

The thermophysical properties of a monolayer graphene sheet were computed using the optimized parameters of the TersoffCG potential. To generate the CG graphene model, the atomistic bond distance (C-C) of the graphene model was changed to the CG bond distance (C-C) using Visual Molecular Dynamics (VMD) [71]. The size of the graphene sheet was 20 × 40 nm^2^, with the armchair and zigzag directions represented by the *x* and *y* directions, respectively, and a thickness of 0.335 nm.

**Figure 11:**
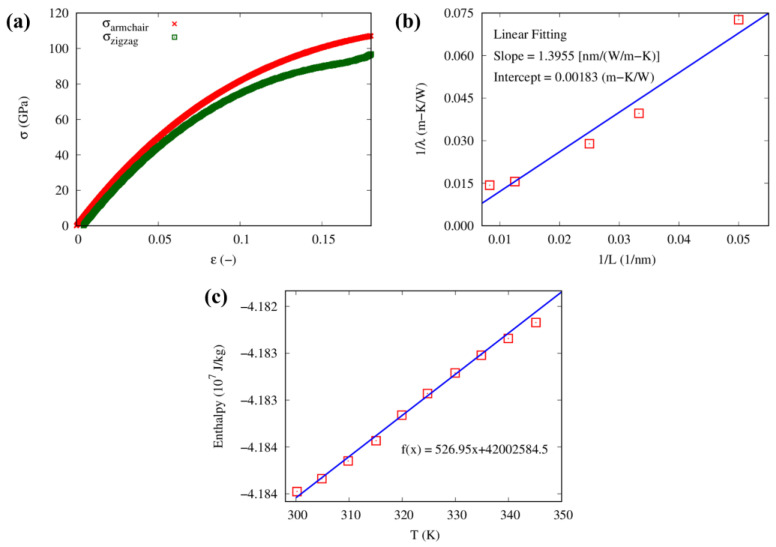
(**a**) Stress vs. strain response of single CG graphene sheet in the armchair and zigzag direction, (**b**) inverse of thermal conductivity-inverse of length curve in armchair direction, and (**c**) enthalpy-temperature plot, with best fitting function.

The system was equilibrated considering an NVT ensemble at 300 K for 100 ps before a uniaxial tension deformation was applied along the armchair and zigzag directions. The stress-strain curve in Figure 11a was considered to calculate the elastic modulus and Poisson’s ratio of the CG graphene in each direction. In the zigzag direction, the elastic modulus and Poisson’s ratio were found to be 981 GPa and 0.14, respectively; while in the armchair direction, they were found to be 1009 GPa and 0.16, respectively. These results agree with previous mesoscopic simulation values 900–1050 GPa (elastic modulus) and 0.14–0.19 (Poisson ratio) [45,48,72].

The thermal conductivity of the monolayer CG graphene model was then investigated using NEMD simulation for different sample lengths ranging from 20 nm to 100 nm. The relationship between the inverse length (1/L) and the inverse thermal conductivity $\left(1/\lambda \right)$ is shown in Figure 11b. In general, the ballistic-to-diffusive crossover formula describes the relationship between length and thermal conductivity [73] as follows:

(7) $$\frac{1}{\lambda \left(L\right)}=\frac{1}{\lambda_{0}}\left(1+\frac{L}{L}\right),$$

where L represents the mean free path of a phonon in graphene, and $\lambda_{0}$ the thermal conductivity with an infinite length. The thermal conductivity values for the armchair and zigzag directions were determined using the inverse intercept from the linear fitting of the CG MD trajectories and were found to be 546 and 520 W/mK, respectively [73]. The specific heat capacity of the CG graphene filler was also computed from the enthalpy and temperature plot shown in Figure 11c. The resulting plot was linearly fitted, and its slope was found to be 526 J/kgK, whereas the experimentally measured $C_{P}$ of graphene is typically 700 J/kgK at room temperature [74].

The CG MD value of thermal conductivity of an infinitely long graphene sheet exhibits lower values compared to the experimental ones of high-quality graphene, which typically range from 3000 to 4000 W/mK [74,75,76]. Computational studies using MD simulations with the AIREBO potential have reported a thermal conductivity of 2600 W/mK [77]. Zhang et al. [78] investigated the thermal conductivity of monolayer graphene using the Tersoff force field. They kept the width fixed at 10 nm and varied the length from 100 nm to 600 nm. The thermal conductivity of graphene with a length of 100 nm was found to be 368 W/mK, while at a length of 600 nm, it increased to 564 W/mK. Similarly, Cao et al. [79] studied the thermal conductivity of monolayer graphene using the Tersoff force field in the length range of 250 nm to 2000 nm and found an approximately constant value of 750 W/mK within this range. Sun et al. [80] employed the Tersoff force field to determine the thermal conductivity of monolayer graphene with lengths ranging from 20 nm to 100 nm. Extrapolation of the data yielded thermal conductivity values of 848 W/mK for the armchair direction and 986 W/mK for the zigzag direction. It is evident that the choice of potential significantly affects the modeled thermal conductivity: based on previous studies, it can be concluded that the Tersoff potential underestimates the thermal conductivity of graphene compared to the AIREBO one.

Overall, it should be noted that the TersoffCG force field is more suitable for the prediction of mechanical properties, while thermal properties are modeled with lower accuracy. The difference between the predicted CG and atomistic values can be attributed to the fact that material thermal behavior is based on phonon transfer, which can be altered when fewer degrees of freedom are considered in the CG model [81]. Therefore, accurately predicting phonon or thermal phenomena can be a daunting task at a coarser level. A tabulated comparison between mesoscopic model predictions, as well as literature measures for the thermophysical properties of the monolayer graphene sheet, are reported in Supplementary Note S5.

### 3.5. Coarse-Grained and Continuum Simulation of Epoxy/Gr Composites

After analyzing the epoxy and graphene CG models separately, these CG MD models were employed to investigate the impact of graphene inclusions on the thermophysical characteristics of the resulting epoxy composites. To achieve this, nanocomposite systems were built, and CG MD simulations were performed using LAMMPS. Various concentrations of graphene (0.5 wt.%, 0.8 wt.%, 1.0 wt.%, 1.5 wt.%, and 2.0 wt.%) were introduced into the epoxy resin. DGEBA, DICY, DETA, and Gr molecules were randomly seeded into a cubic box using LAMMPS, followed by a multistep cross-linking process. Equilibration procedures were performed, including energy minimization, using NVT and NPT ensembles at a temperature of 300 K and pressure of 1 atm, and a time step of 1 fs. The system was equilibrated after 4–5 ns, and the equilibrated systems were then used to determine the thermophysical properties of the resulting nanocomposite. To facilitate comparison with previous results, a cross-linking degree of 70% was considered for the epoxy matrix. Figure 6b illustrates the equilibrated 70% cross-linked system of the CG epoxy/Gr nanocomposite.

The input parameters for the RVE continuum models to be compared were obtained from the CG simulation results, including the parameters for epoxy such as density (1.24 g/cm^3^), elastic constant (1.419 GPa), Poisson’s ratio (0.3), specific heat capacity (550.60 J/kgK), and thermal conductivity (0.099 W/mK), as well as the graphene density (1.28 g/cm^3^), elastic constant (916 GPa), Poisson’s ratio (0.14), specific heat capacity (526.95 J /kgK), and thermal conductivity (13.95 W/mK for the simulated length). The RVE model was created by assigning the same mass fraction (0.5 wt.%, 0.8 wt.%, 1.0 wt.%, 1.5 wt.%, and 2.0 wt.%) to the CG model.

**Figure 12:**
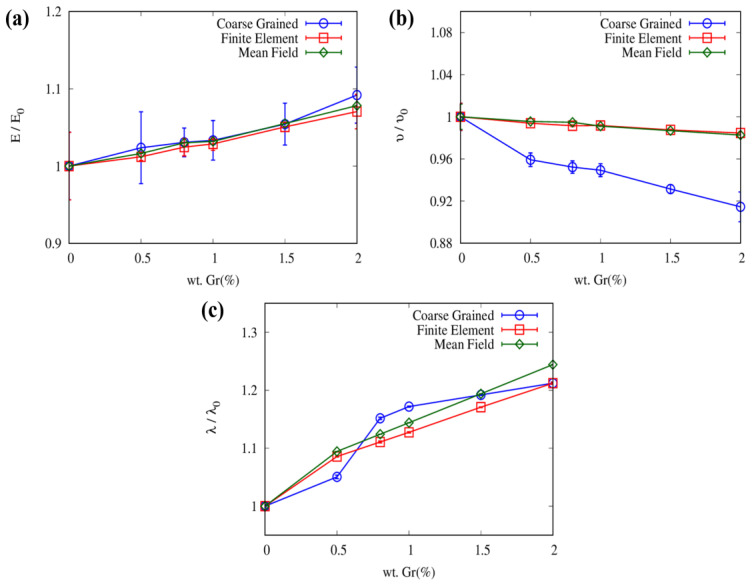
Average values with a standard deviation of (**a**) elastic modulus, (**b**) Poisson’s ratio, and (**c**) thermal conductivity enhancement of epoxy resin reinforced with graphene nanofiller. Quantities computed for the nanocomposites are normalized with respect to neat epoxy ones (subscript 0). Results from Coarse-Grained (CG), Finite Element (FE), and Mean Field (MF) models are reported. Each sample was tested in x, y, and z directions and results were averaged.

Figure 12a illustrates the enhanced elastic modulus of nanocomposite (*E*) with respect to that of pristine epoxy (*E_0_*) as a function of the graphene weight fraction for CG, MF, and FEM models. The elastic modulus is enhanced with all models with an increase in the graphene weight fraction. In the CG MD simulations, the elastic modulus increases from 2.32% (0.5 wt.%) to 9.16% (2.0 wt.%), whereas in the FEM simulation, the elastic modulus increases from 1.19% (0.5 wt.%) to 7.04% (2.0 wt.%), compared to the neat epoxy. Both FEM and MF models exhibit a similar trend. Figure 12b presents the Poisson’s ratio of the epoxy/Gr nanocomposite (ν) with respect to that of pristine epoxy ($\nu_{0}$) predicted at the mesoscopic and continuum levels. All models show a decrease in the Poisson’s ratio with an increase in the weight percentage of the graphene, with a steeper decrease observed in the CG model than in the FEM and MF ones.

The effect of graphene on the thermal conductivity of the epoxy/Gr nanocomposite was also investigated, and the results are presented in Figure 12c. Results indicate that as the weight percentage of graphene increases, the thermal conductivity of the epoxy/Gr composite also increases. This is due to the effective phonon transmission provided by the high graphene content, resulting in an increase in the thermal conductivity of the composite material. The CG MD simulation yielded a maximum thermal conductivity enhancement of 21% (2.0 wt.%) with respect to the neat epoxy, which is in close agreement with that predicted by the FEM model. The thermal conductivity of the epoxy matrix composite linearly increases with higher concentrations of graphene fillers. Correspondingly, experimental studies [82,83] also demonstrated a similar trend, where the thermal conductivity of the epoxy matrix composite increases with higher graphene concentration.

Clearly, the size of the simulation domain may significantly impact the measured properties. Employing a larger domain size allows for improved statistical sampling, leading to more precise and reliable outcomes. Tsige et al. [84] observed an increase in the glass transition temperature as the system size of epoxy resin decreased, ranging from 35,000 to 2000 atoms. In contrast, Strachan et al. [22,85] observed that relatively larger systems (16,000 to 65,000 atoms) had a weak size effect on the glass transition temperature and mechanical characteristics of the simulated epoxy resin. Similarly, Feng et al. [86] examined the effect of length on the thermal conductivity in polymers using MD simulations, finding that thermal conductivity is weakly affected by size for lengths greater than 50 Å. Based on these observations, our study employed systems consisting of approximately 20,000 beads for both the epoxy resin and epoxy/Gr composite. These systems exhibited dimensions exceeding 100 × 100 × 100 Å^3^, coherently with previous investigations employing atomistic as well as CG MD models of epoxy resin [87,88,89,90,91,92].

Overall, at least for the considered nanocomposites, both mesoscopic and continuum models provide consistent enhancements in the thermophysical properties of neat epoxy when graphene fillers are included. A detailed list of all the results is available in Supplementary Note S5.

## 4. Conclusions

This study employed multiscale computational techniques to investigate the impact of graphene on the thermophysical properties of epoxy matrix composite. By integrating atomistic, mesoscopic, and continuum models, we accurately predicted properties including density, glass-transition temperature, elastic modulus, Poisson’s ratio, and thermal conductivity. Initially, a full atomic model of the epoxy resin was constructed using a COMPASS force field, yielding precise predictions for various properties. However, the specific heat capacity was found to be overestimated compared to the experimental values.

Subsequently, a mesoscopic (coarse-grained) force field was developed using the iterative Boltzmann inversion (IBI) method, based on the atomistic model. The developed mesoscopic model exhibited significant agreement with the atomistic and experimental values for density, glass-transition temperature, and Poisson ratio. However, the predicted values for Young’s modulus and thermal conductivity were 47% and 40% lower than the atomistic values, respectively. The specific heat capacity was also underestimated compared to the experimental values. For graphene, a coarse-grained force field based on the TersoffCG potential was employed, with optimized parameters for mechanical characteristics. This model demonstrated good agreement with published MD and experimental results for the mechanical properties than the thermal conductivity.

Furthermore, the developed mesoscopic models were utilized to determine the thermophysical properties of epoxy–graphene nanocomposites and compared them to similar continuum models, showing consistent results. The findings indicated that the increasing weight percentage of graphene-enhanced elastic modulus and thermal conductivity while reducing the Poisson’s ratio. Despite the coarse-grained model having lower prediction accuracy compared to full atomistic models, its 10× simulation speed enables the modeling of material sizes beyond classical MD simulations. In the future, this approach could be extended to investigate the thermophysical behavior of epoxy resin systems with other carbon-based nanoparticles, such as graphene oxide, reduced graphene oxide, and carbon nanotubes.

## Data Availability

Data associated to this work are available in the Supplementary Materials. Further data presented in this study are available on request from the corresponding author.

## Supplementary Materials

The following supporting information can be downloaded at https://www.mdpi.com/article/10.3390/nano13131960/s1: Supplementary Notes S1: Crosslinking mechanism; Supplementary Notes S2: Mesoscopic model of graphene; Supplementary Notes S3: Epoxy resin CG force field (EPO-CGff); Supplementary Notes S4: Epoxy graphene composites CG force field (EPO-GRP-CGff); Supplementary Notes S5: Tabulated results. References [20,56,57,58,59,60,61,62,63,64,65,66,74,76,77,80,93,94,95,96] are cited in the Supplementary Materials.
