# Supplementary material for: Atomistic to Mesoscopic Modelling of Thermophysical Properties of Graphene-Reinforced Epoxy Nanocomposites
Source: Nanomaterials (Basel). 2023 Jun 28;13(13):1960. doi: 10.3390/nano13131960 (PMC10343328; doi:10.3390/nano13131960)
Supplement: Supplementary file 1 [file nanomaterials-13-01960-s001.zip › nanomaterials-2447595-supplementary.pdf]

Supplementary Materials

Atomistic to Mesoscopic Modelling of Thermophysical Properties of Graphene-Reinforced Epoxy Nanocomposites

Supplementary Note S1: Crosslinking mechanism

i. Atomistic model of epoxy

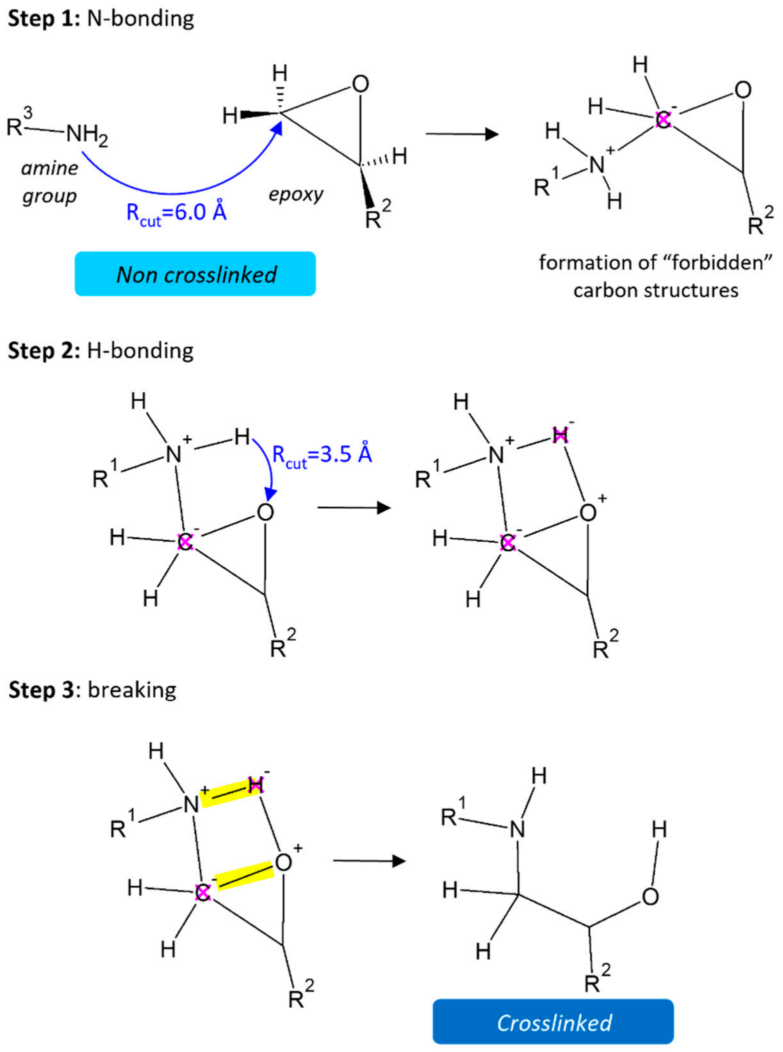

Figure S1. Cross-linking protocol for hardeners containing amine groups.

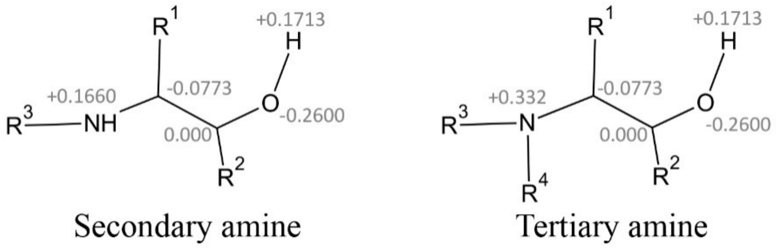

Figure S2. Charge update (Δq) of atoms after cross-linking process (values in eV).

ii. Mesoscopic model of epoxy

Polymer	Type of bead	Bead ID*	Description of bead
**Epoxy resin (EPO)**	E	1	Beads containing unreacted epoxy group in DGEBA
	G	2	Oxyphenylene groups in DGEBA
	B	3	Bead containing central dimethyl group in DGEBA
	Ad	4	Primary amine group in DETA
	Ae	5	Secondary amine group in DETA
	Ay	6	Amine group in DICY
	Y	7	Non-amine group in DICY
	E’	8	Beads containing reacted epoxy group in DGEBA
	Ad1	9	Primary amine group in DETA – reacted once
	Ad2	10	Primary amine group in DETA – reacted twice
	Ae1	11	Secondary amine group in DETA – reacted
	Ay1	12	Amine group in DICY – reacted once
	Ay2	13	Amine group in DICY – reacted twice

Table S1. List of beads for coarse-grained (CG) model of epoxy resin.

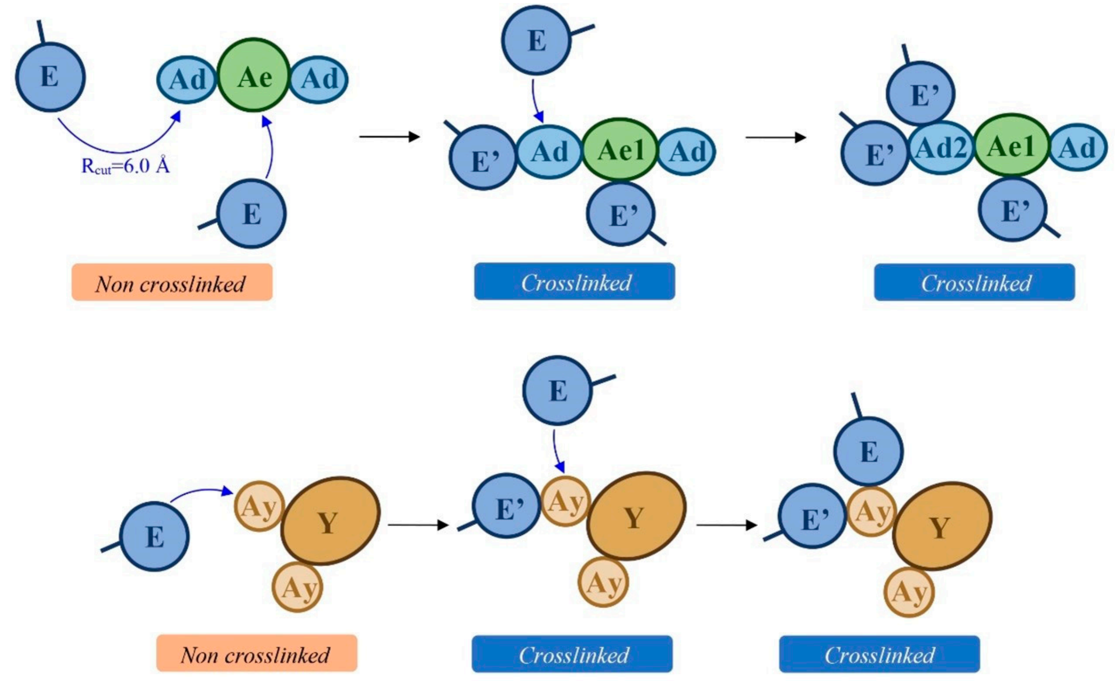

Figure S3. Cross-linking mechanism of CG beads.

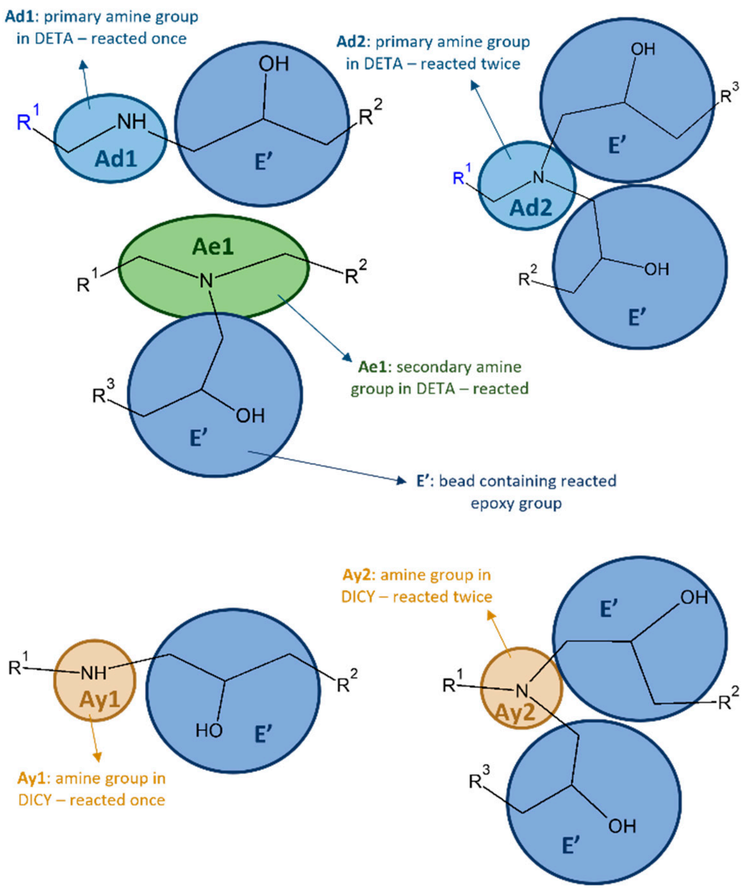

Figure S4. Mapping scheme for cross-linked groups of epoxy resin.

Supplementary Note S2: Mesoscopic model of graphene

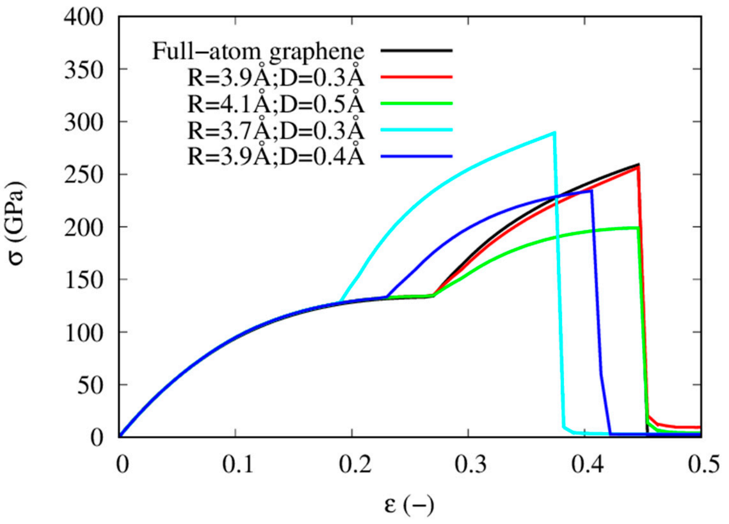

Figure S5. Optimization of the values of R and D for the TersoffCG potential.

Parameter	Tersoff potential	TersoffCG potential
m	3	3
γ	1	1
$\lambda_3(Å^{-1})$	0	0
c	38049	38049
d	4.3484	4.3484
$\cos\theta_0$	-0.57058	-0.57058
n	0.72751	0.72751
β	0.00000015724	0.00000015724
$\lambda_2(Å^{-1})$	2.2119	1.10595
B(kcal/mol)	7995.2	31980.8
R(Å)	1.95	3.90
D(Å)	0.15	0.30
$\lambda_1(Å^{-1})$	3.4879	1.74395
A(kcal/mol)	32137.67024	128550.68096

Table S2. Parameters of Tersoff and TersoffCG potentials for graphene.

Supplementary Note S3: Epoxy resin CG force field (EPO-CGff)

Bond	Angle			
E – G	E – G – B	E' – Ae1 – Ad1	E' – G – B	Ad – Ae1 – Ad2
G – B	G – B – G	E' – Ae1 – Ad2	Ay2 – Y – Ay	Ad1 – Ae – Ad
Ad – Ae	AY – Y – Ay	G – E' – Ad1	Ay1 – Y – Ay	Ad2 – Ae1 – Ad2
Ay – Y	Ad – Ae – Ad	G – E' – Ad2	Ay1 – Y – Ay1	Ad – Ae – Ad2
E' – Ad1	E' – Ad1 – Ae	G – E' – Ae1	Ay1 – Y – Ay2	Ad1 – Ae1 – Ad2
E' – Ad2	E' – Ad1 – Ae1	E' – Ay1 – Y	Ay2 – Y – Ay2	Ad2 – Ae – Ad1
E' – Ae1	E' – Ad2 – Ae	E' – Ay2 – Y	E' – Ad2 – E'	Ad – Ae1 – Ad1
E' – Ay1	E' – Ad2 – Ae1	G – E' – Ay1	E' – Ay2 – E'	Ad2 – Ae – Ad2
E' – Ay2	E' – Ae1 – Ad	G – E' – Ay2	Ad – Ae1 – Ad	

Table S3. List of potentials in the developed EPO-CGff force field for bonded interactions (bonds and angles).

Non-bonded				
E ··· E	E ··· Y	G ··· Y	Ad ··· Ad	Ae ··· Y
E ··· G	G ··· G	B ··· B	Ad ··· Ae	Ay ··· Ay
E ··· B	G ··· B	B ··· Ad	Ad ··· Ay	Ay ··· Y
E ··· Ad	G ··· Ad	B ··· Ae	Ad ··· Y	Y ··· Y
E ··· Ae	G ··· Ae	B ··· Ay	Ae ··· Ae	
E ··· Ay	G ··· Ay	B ··· Y	Ae ··· Ay	

Table S4. List of potentials in the developed EPO-CGff force field for non-bonded interactions.

i. Bond potentials

Bond	Potential
**E – G**	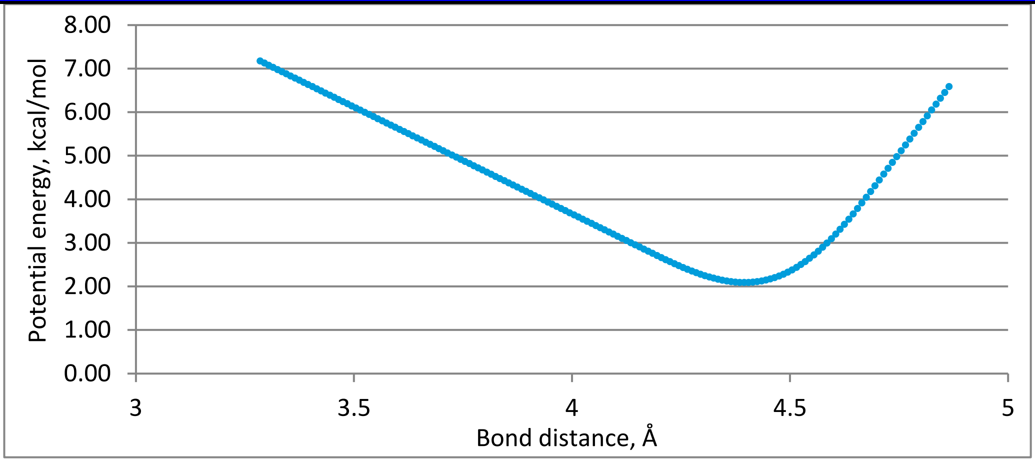

<table>
<tr><th>Bond</th><th>Potential</th></tr>
<tr><td>G – B</td><td>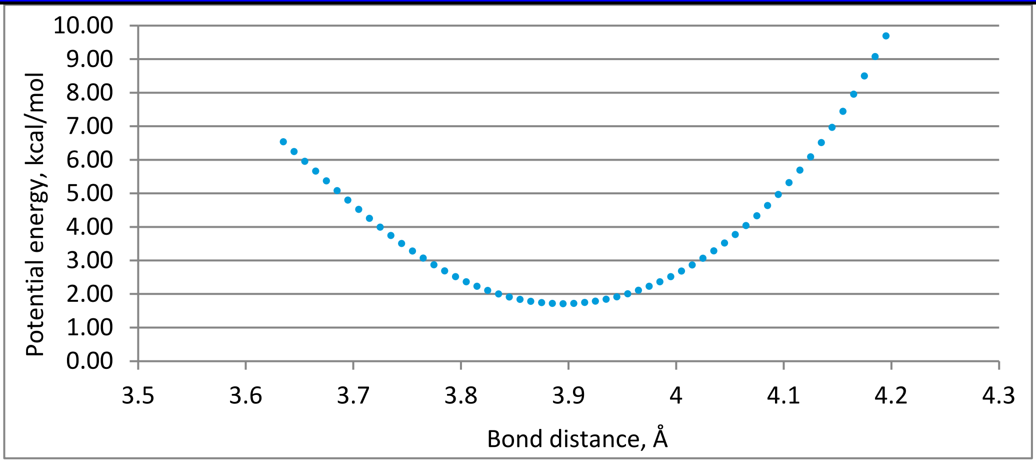
</td></tr>
<tr><td>Ad – Ae</td><td>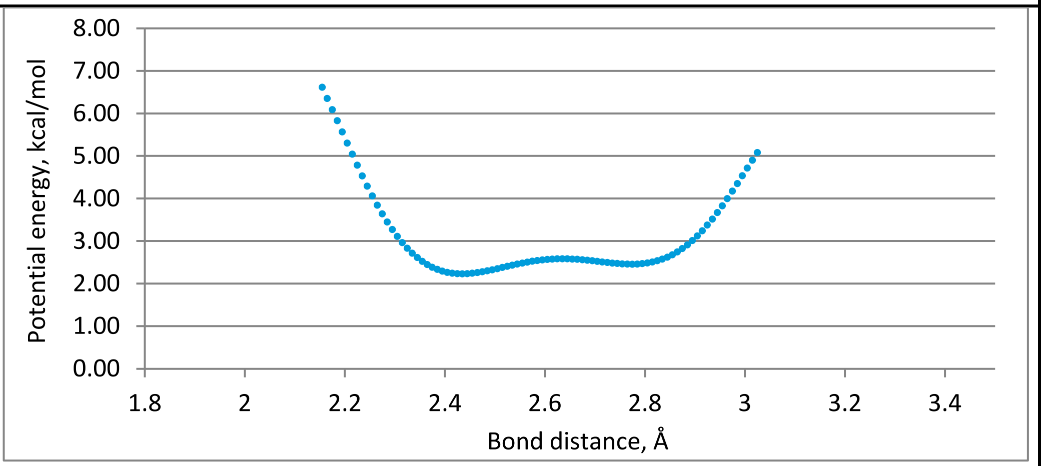
</td></tr>
<tr><td>Ay – Y</td><td>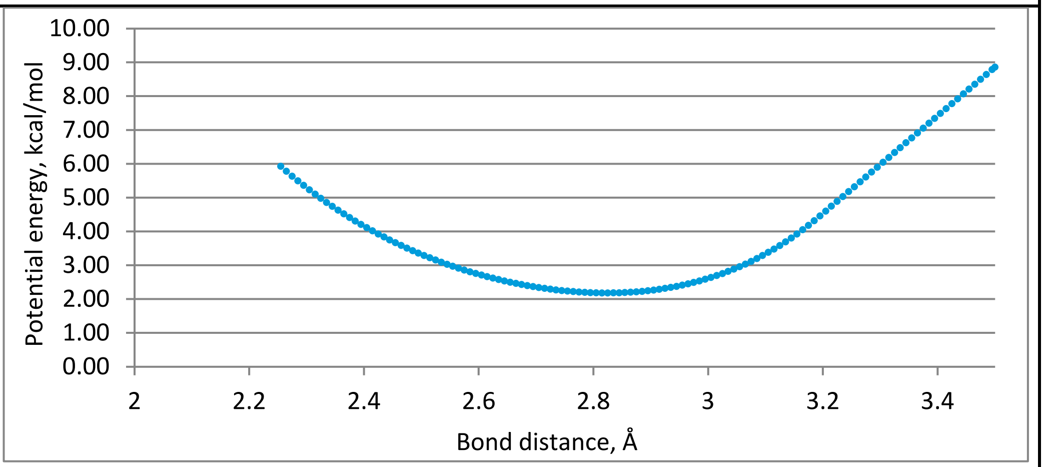
</td></tr>
</table>

<table>
<tr><th>Bond</th><th>Potential</th></tr>
<tr><td>E’ – Ad1</td><td>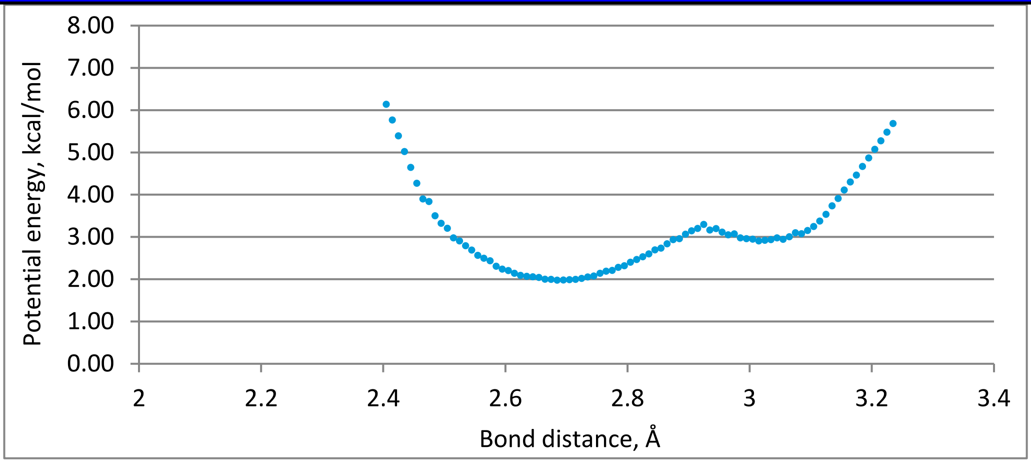
</td></tr>
<tr><td>E’ – Ad2</td><td>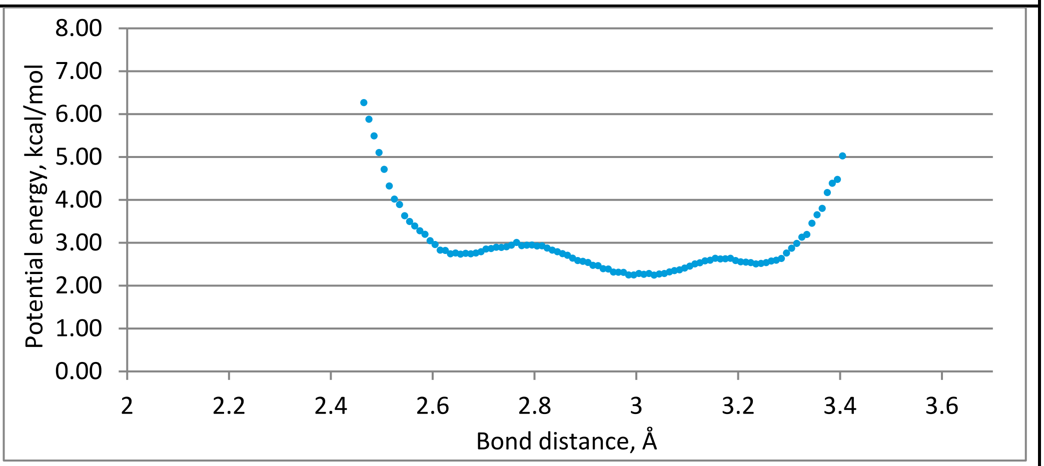
</td></tr>
<tr><td>E’ – Ae1</td><td>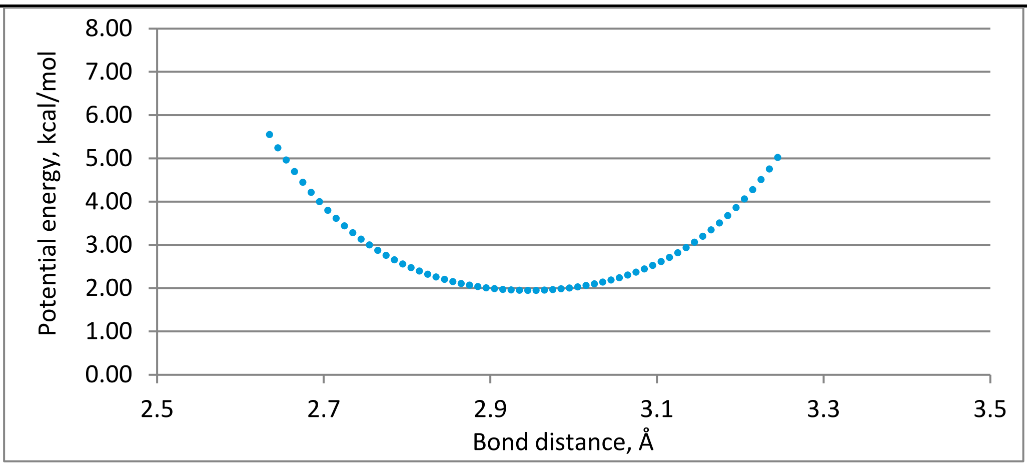
</td></tr>
</table>

Bond	Potential
E’ – Ay1	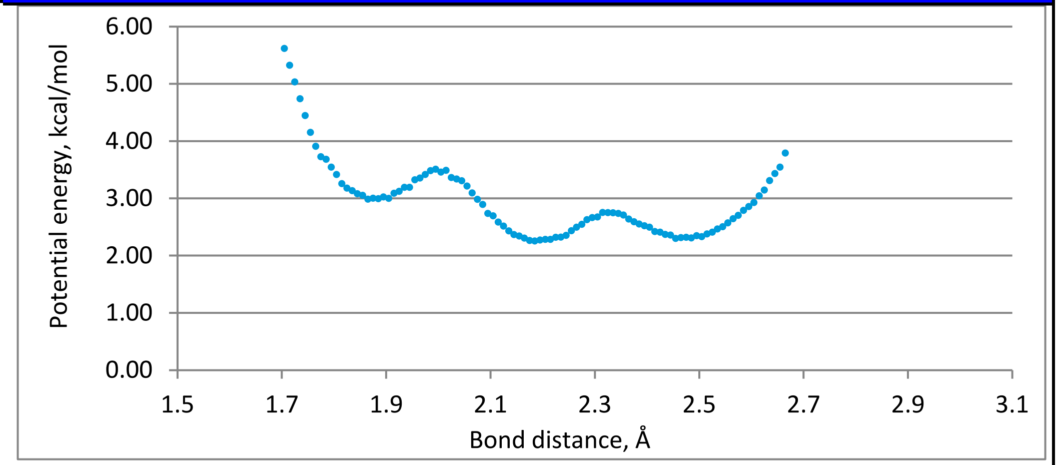
E’ – Ay2	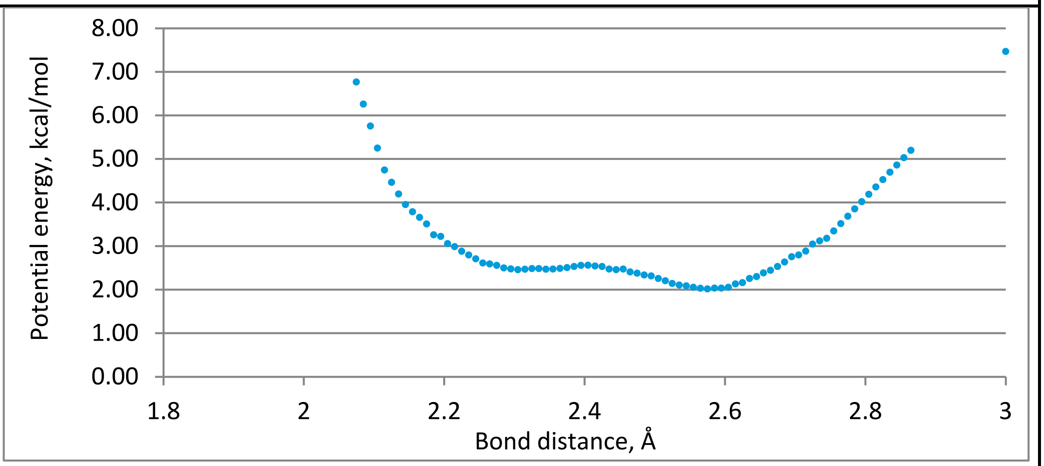

ii. Angle Potentials

Angle	Potential
E – G – B	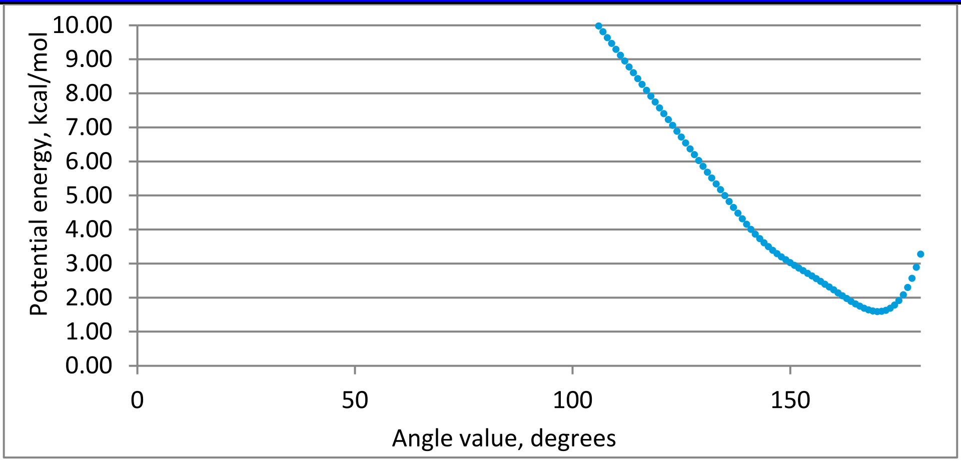

<table>
<tr><th>Angle</th><th>Potential</th></tr>
<tr><td>G – B – G</td><td>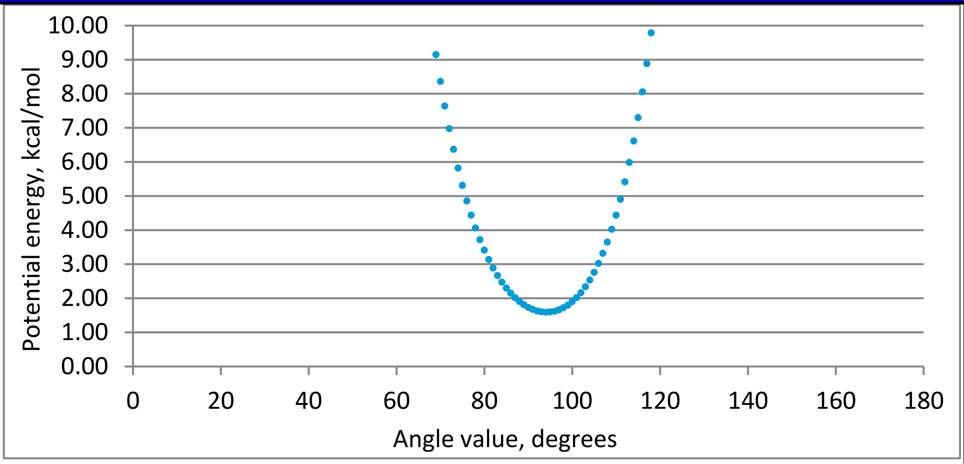
</td></tr>
<tr><td>AY – Y – Ay</td><td>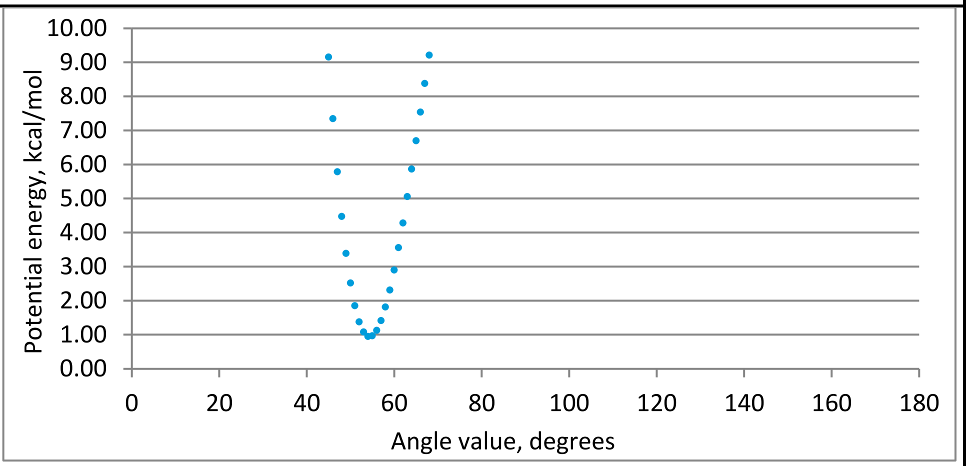
</td></tr>
<tr><td>Ad – Ae – Ad</td><td>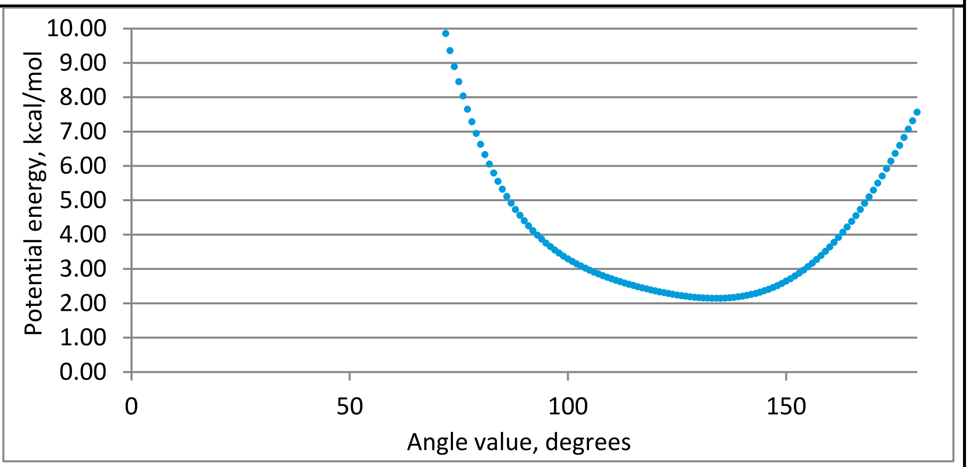
</td></tr>
</table>

Angle	Potential
**E' – Ad1 – Ae**	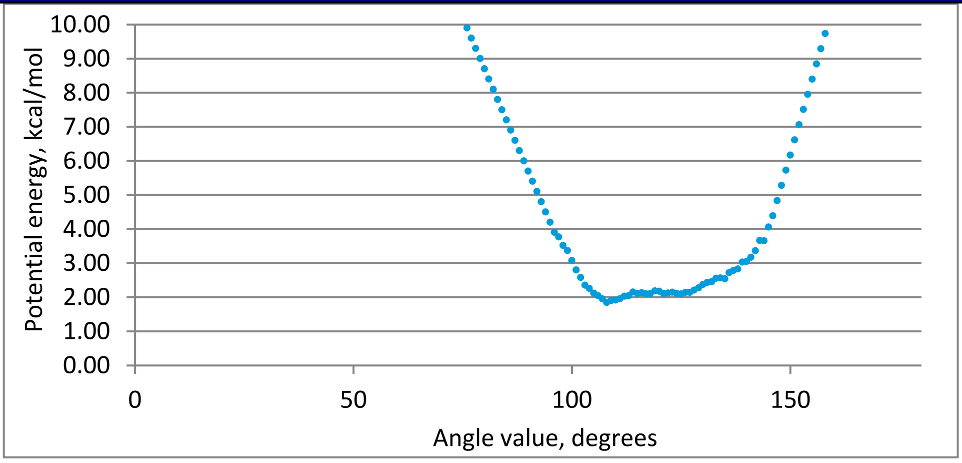
**E' – Ad1 – Ae1**	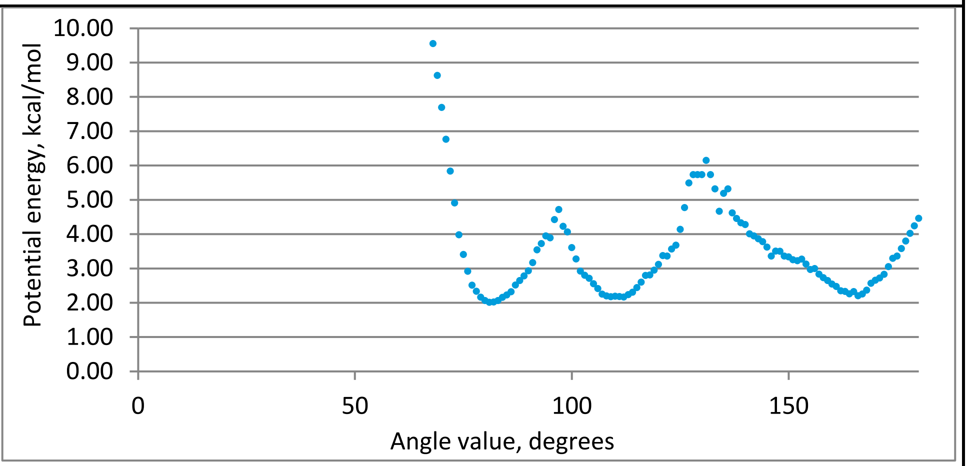
**E' – Ad2 – Ae**	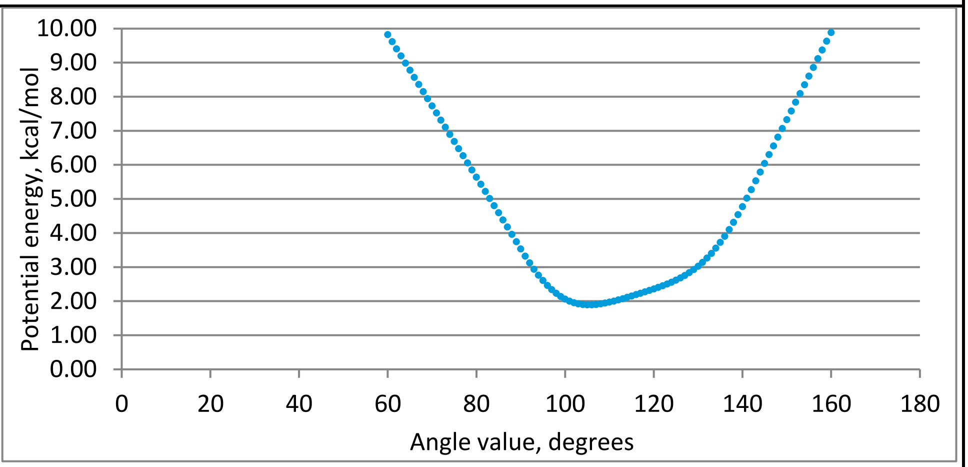

<table>
<tr><th>Angle</th><th>Potential</th></tr>
<tr><td>E’ – Ad2 – Ae1</td><td>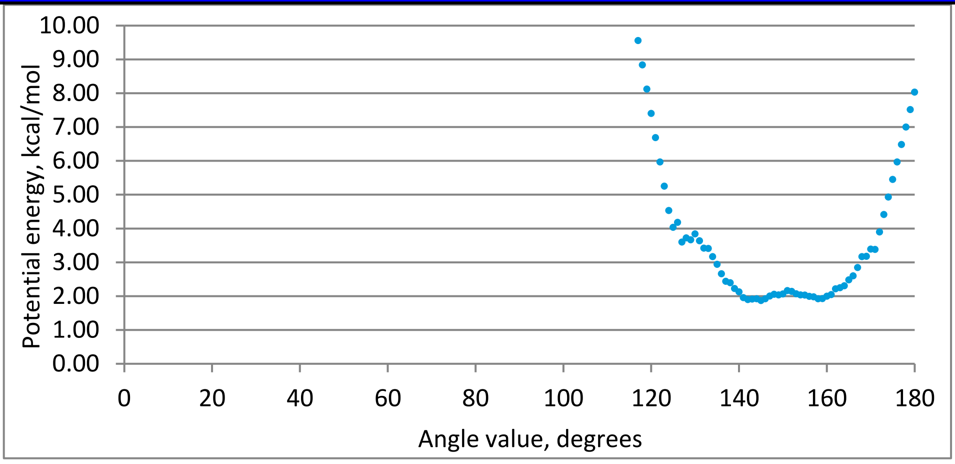
</td></tr>
<tr><td>E’ – Ae1 – Ad</td><td>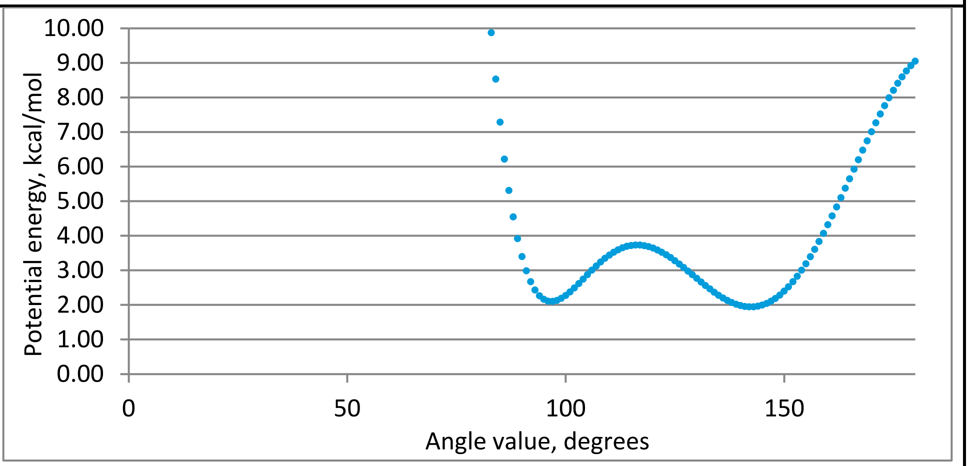
</td></tr>
<tr><td>E’ – Ae1 – Ad1</td><td>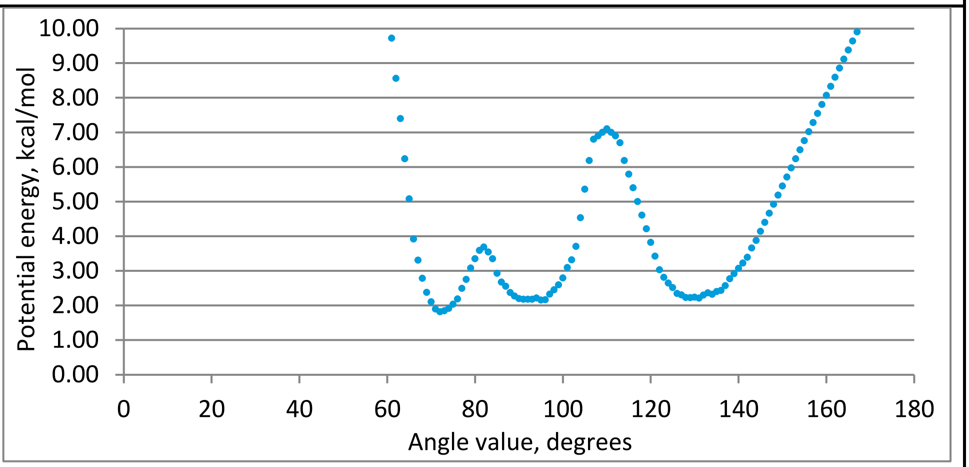
</td></tr>
</table>

Angle	Potential
**E' – Ae1 – Ad2**	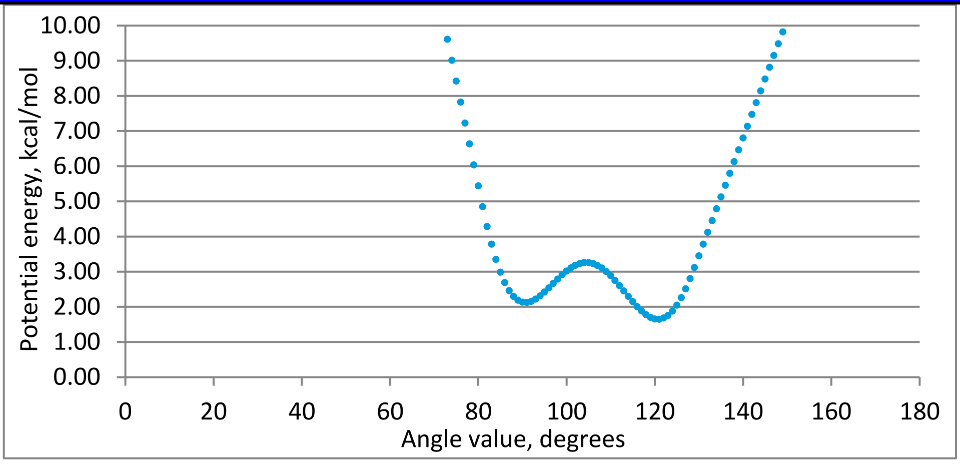
**G – E' – Ad1**	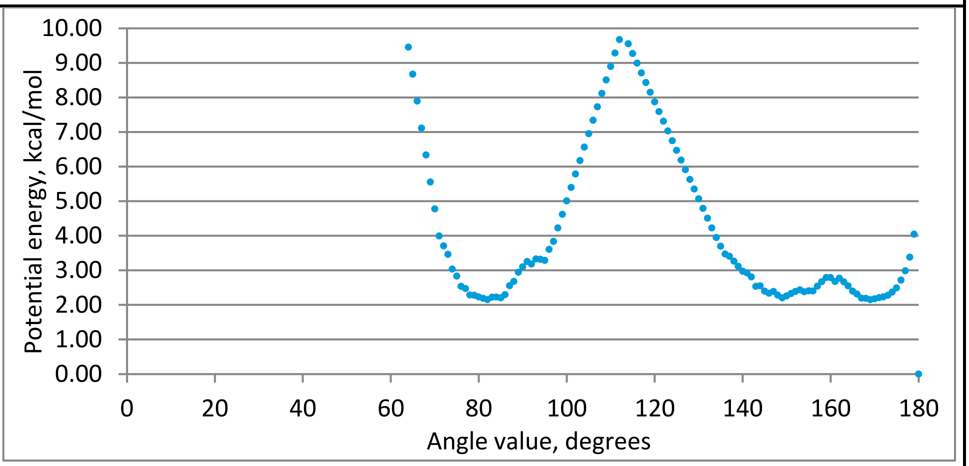
**G – E' – Ad2**	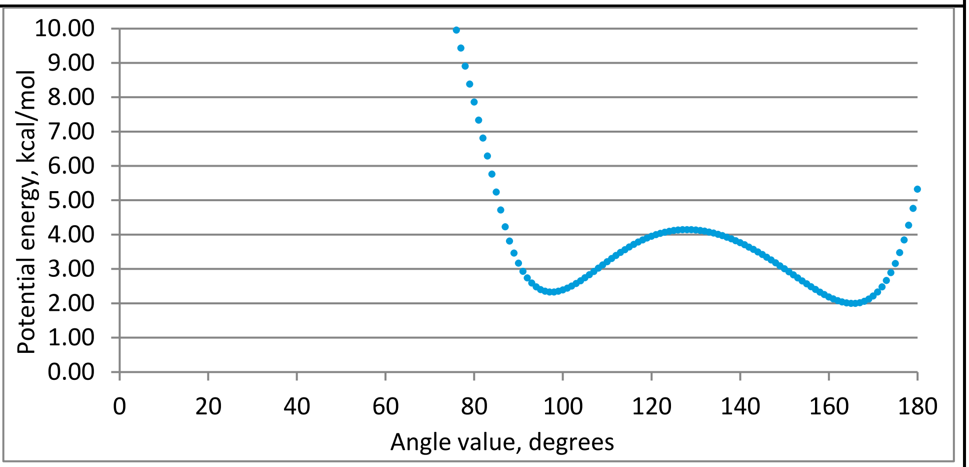

Angle	Potential
**G – E’ – Ae1**	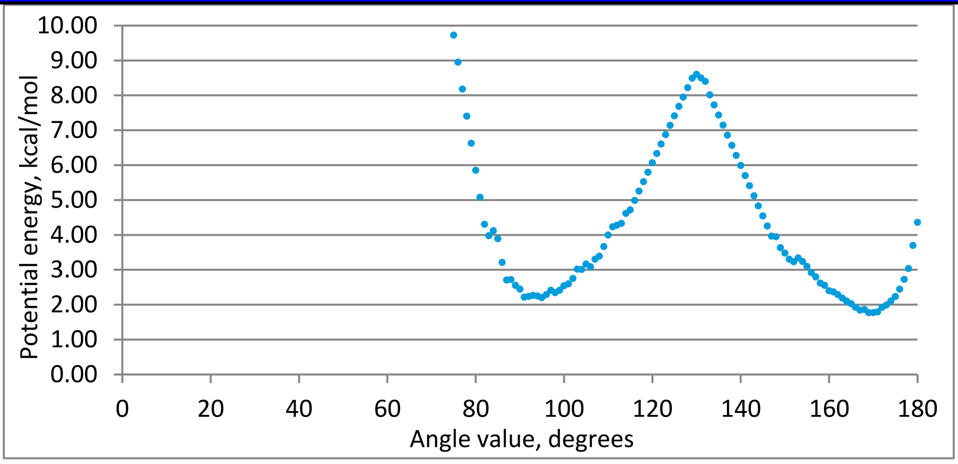
**E’ – Ay1 – Y**	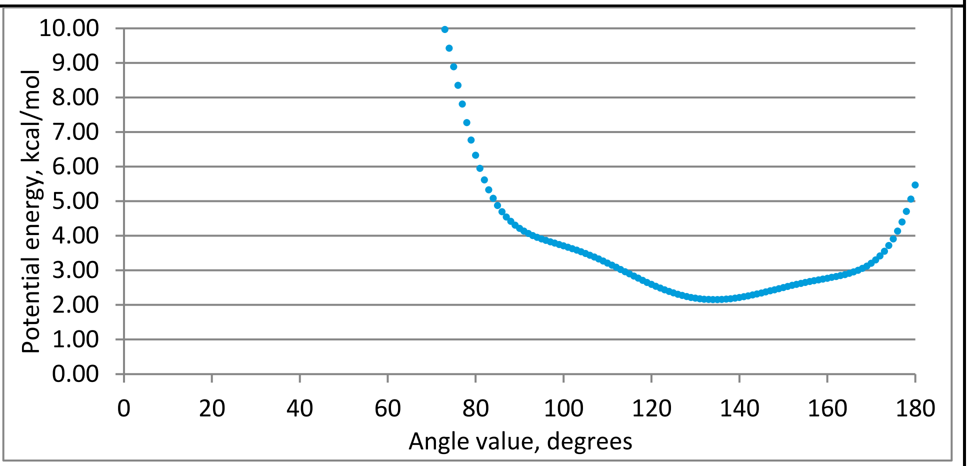
**E’ – Ay2 – Y**	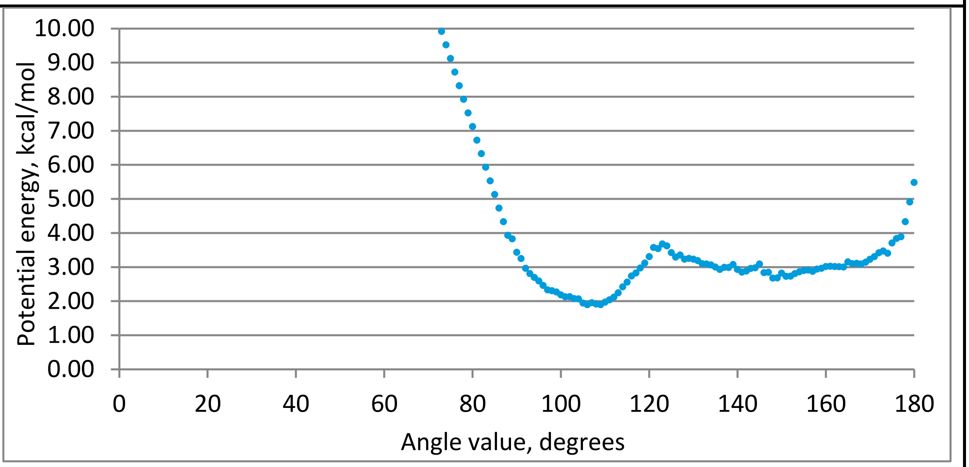

<table>
<tr><th>Angle</th><th>Potential</th></tr>
<tr><td>G – E’ – Ay1</td><td>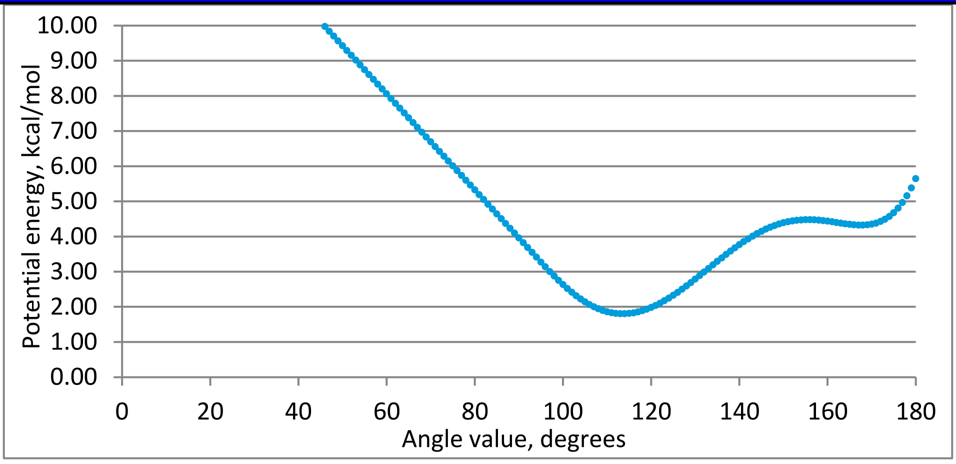
</td></tr>
<tr><td>G – E’ – Ay2</td><td>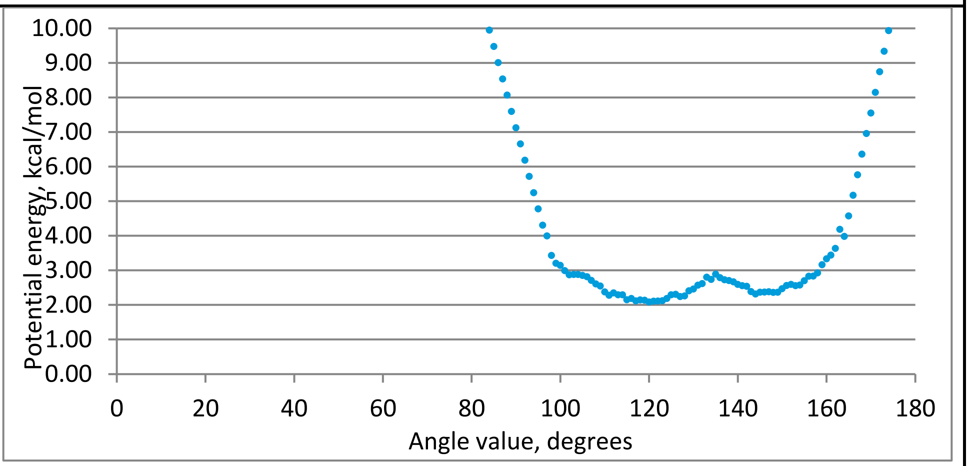
</td></tr>
<tr><td>E’ – G – B</td><td>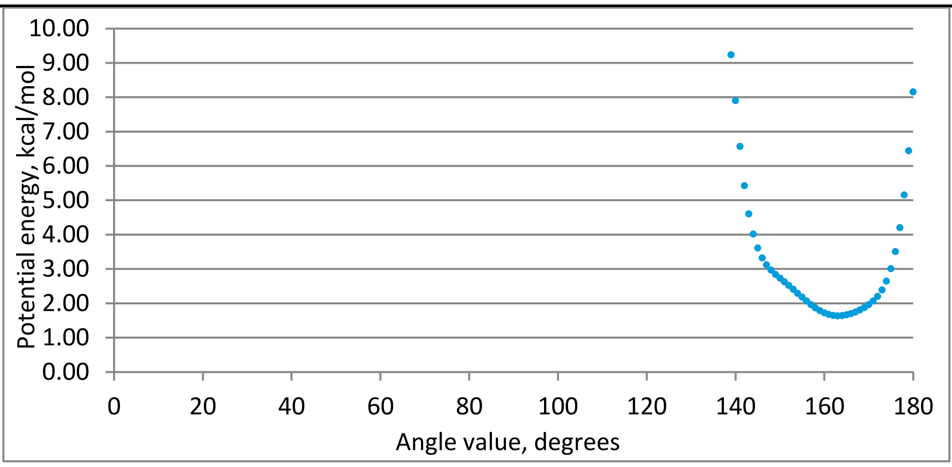
</td></tr>
</table>

Angle	Potential
**Ay2 – Y – Ay**	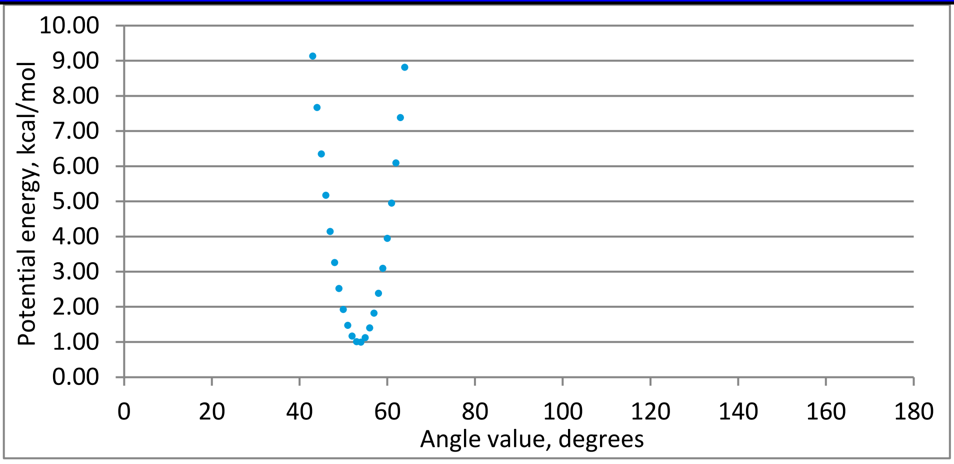
**Ay1 – Y – Ay**	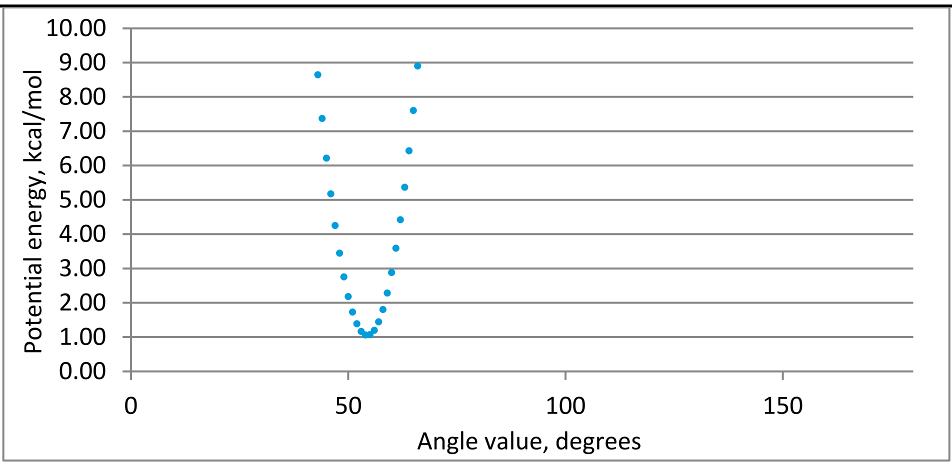
**Ay1 – Y – Ay1**	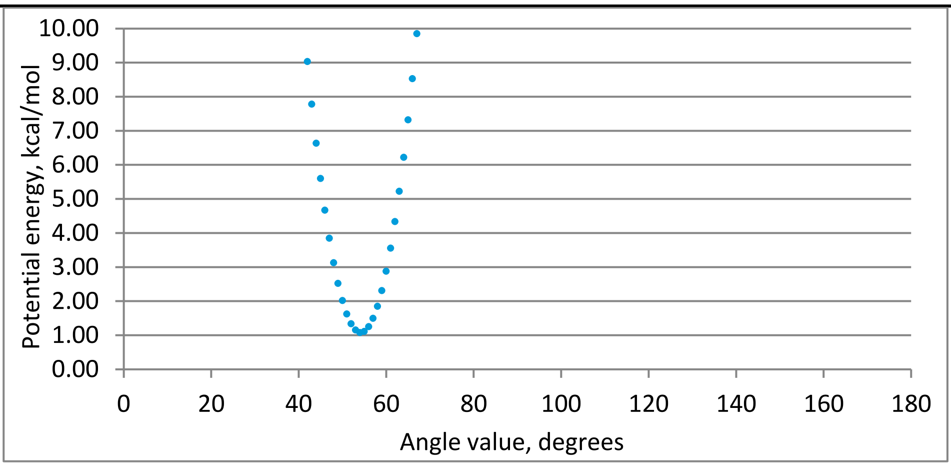

<table>
<tr><th>Angle</th><th>Potential</th></tr>
<tr><td>Ay1 – Y – Ay2</td><td>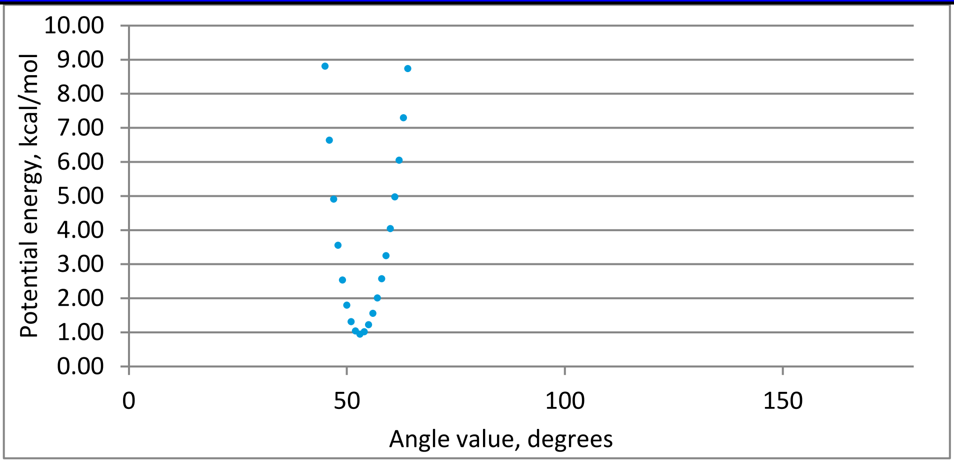
</td></tr>
<tr><td>Ay2 – Y – Ay2</td><td>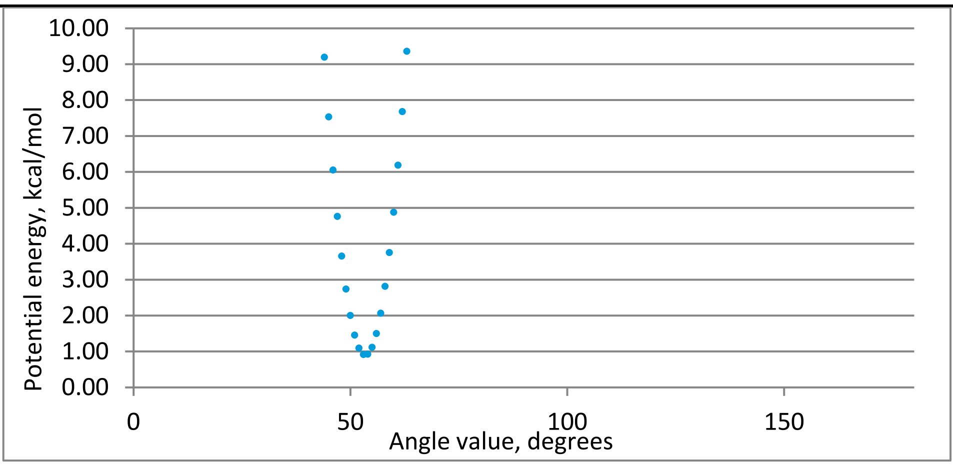
</td></tr>
<tr><td>E’ – Ad2 – E’</td><td>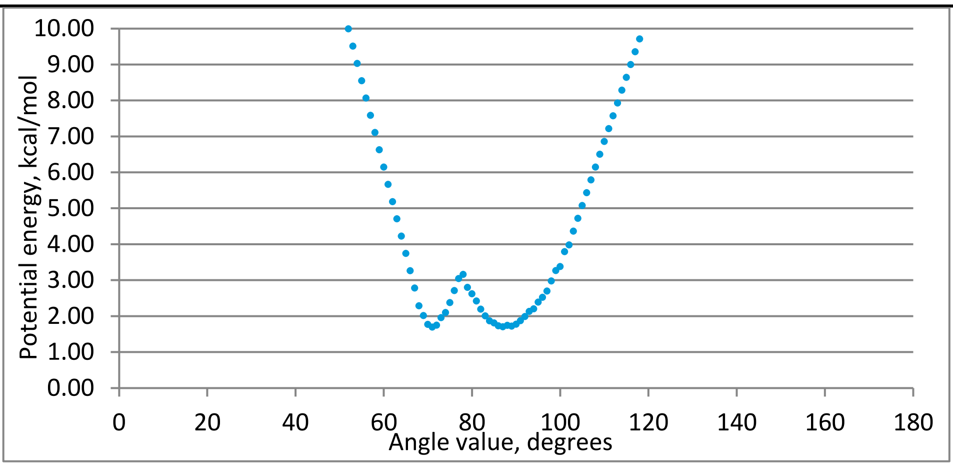
</td></tr>
</table>

<table>
<tr><th>Angle</th><th>Potential</th></tr>
<tr><td>E’ – Ay2 – E’</td><td>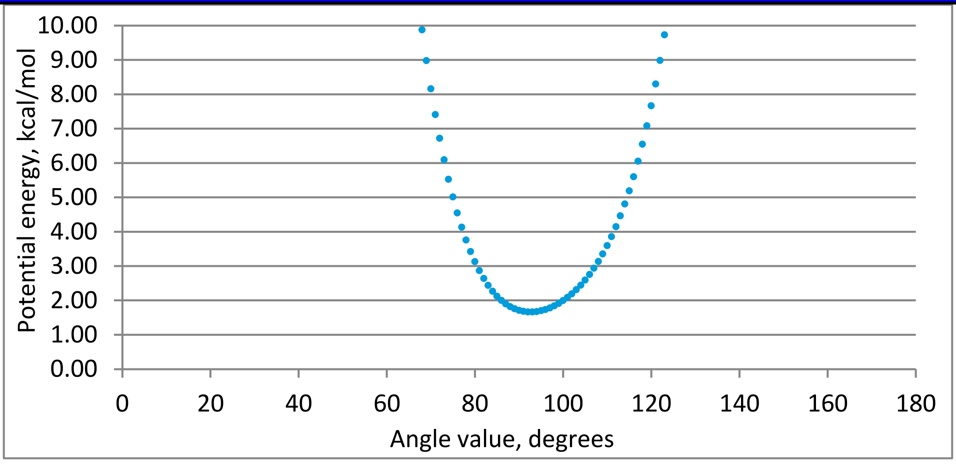
</td></tr>
<tr><td>Ad – Ae1 – Ad</td><td>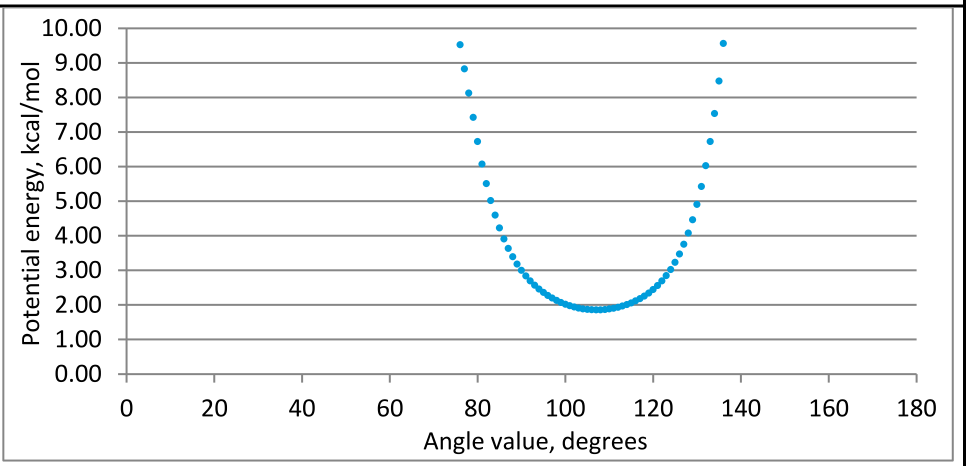
</td></tr>
<tr><td>Ad – Ae1 – Ad2</td><td>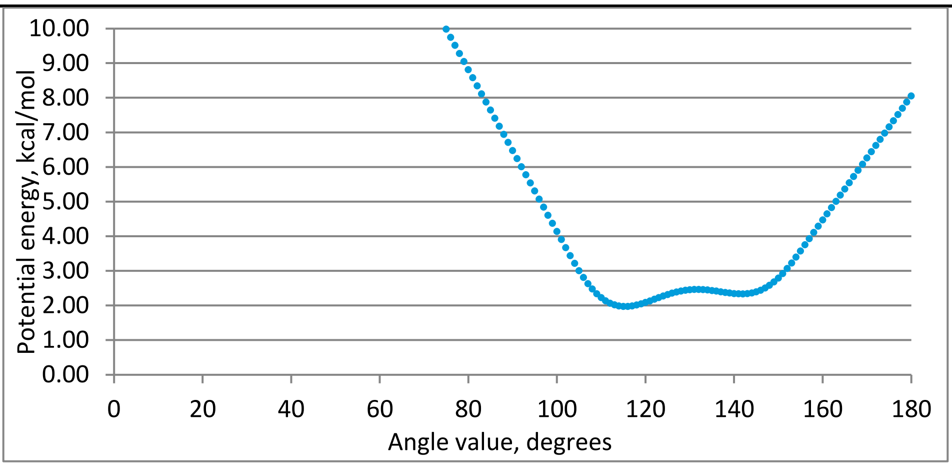
</td></tr>
</table>

Angle	Potential
**Ad1 – Ae – Ad**	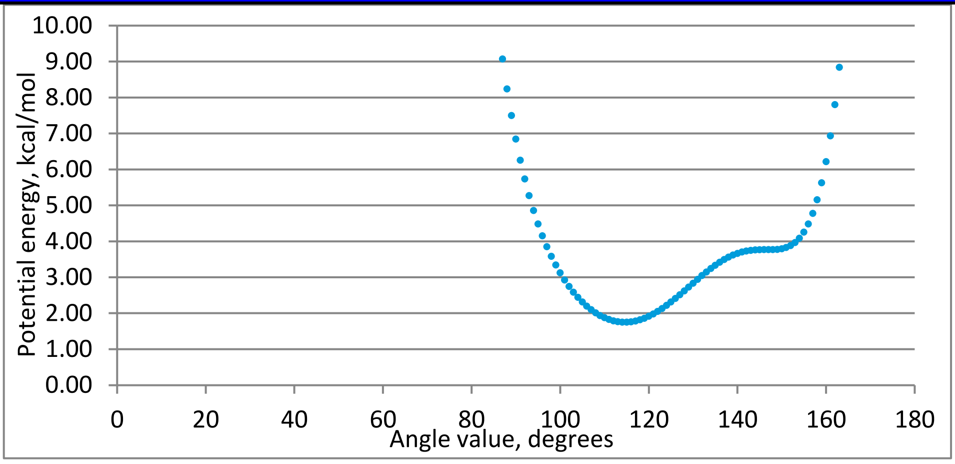
**Ad2 – Ae1 – Ad2**	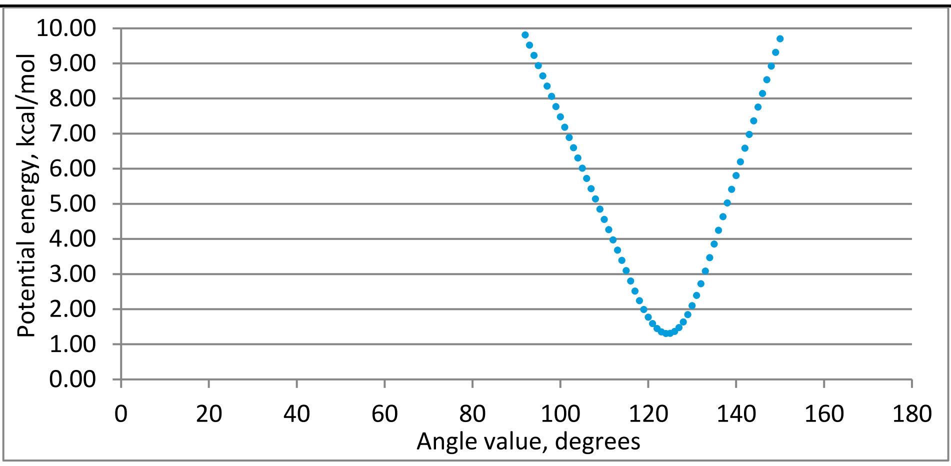
**Ad – Ae – Ad2**	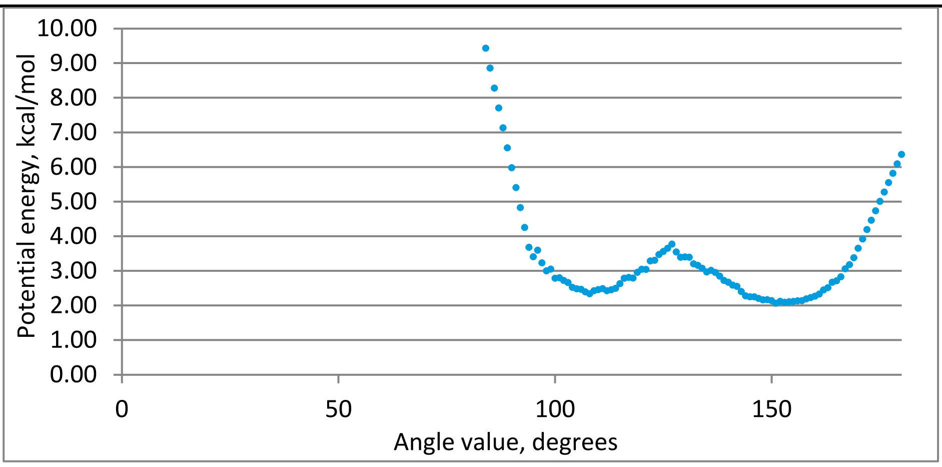

Angle	Potential
**Ad1 – Ae1 – Ad2**	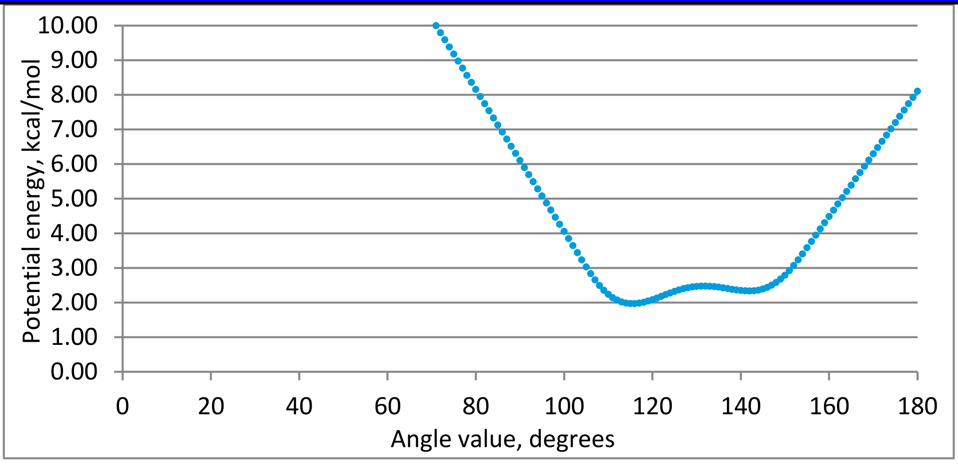
**Ad2 – Ae – Ad1**	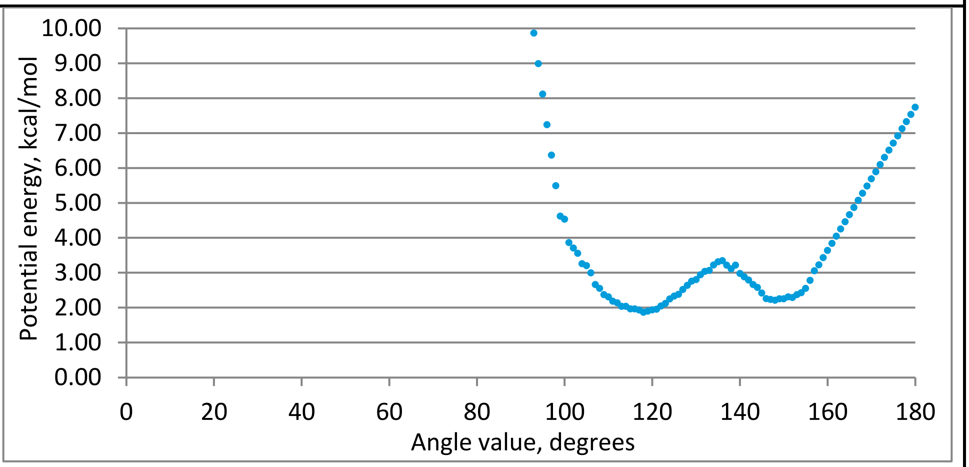
**Ad – Ae1 – Ad1**	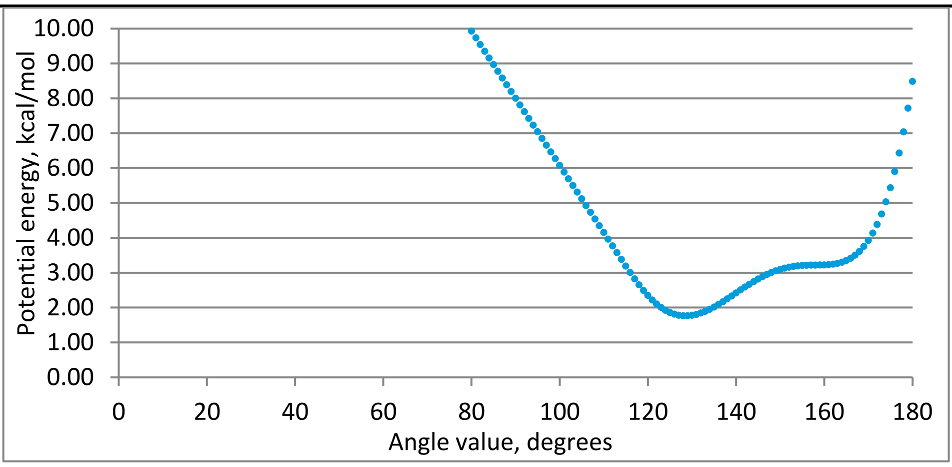

**Angle**	**Potential**
**Ad2 – Ae – Ad2**	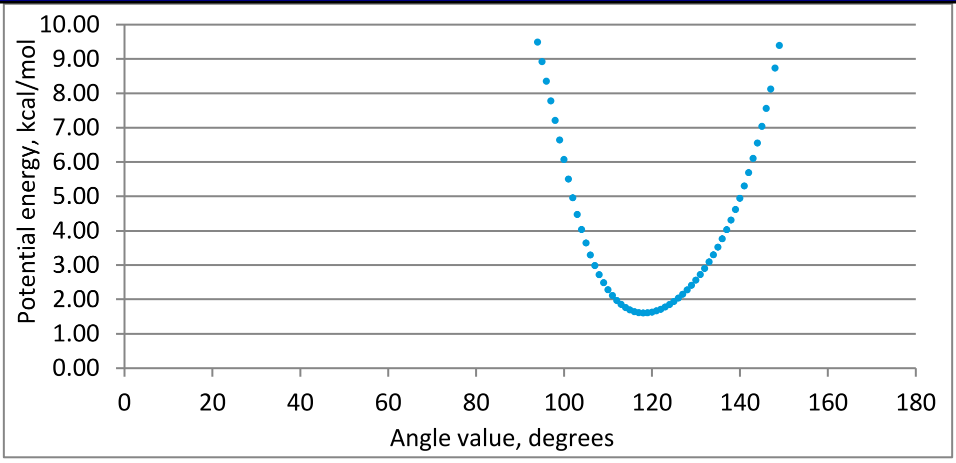

iii. Non-bonded potentials

Interaction	Potential (energy [kcal·mol^{-1}] versus distance [Å])
E···E	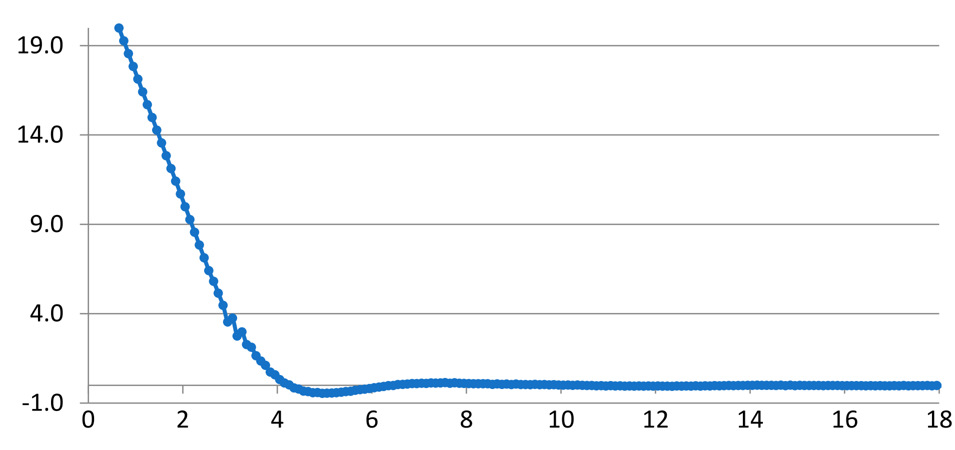
E···G	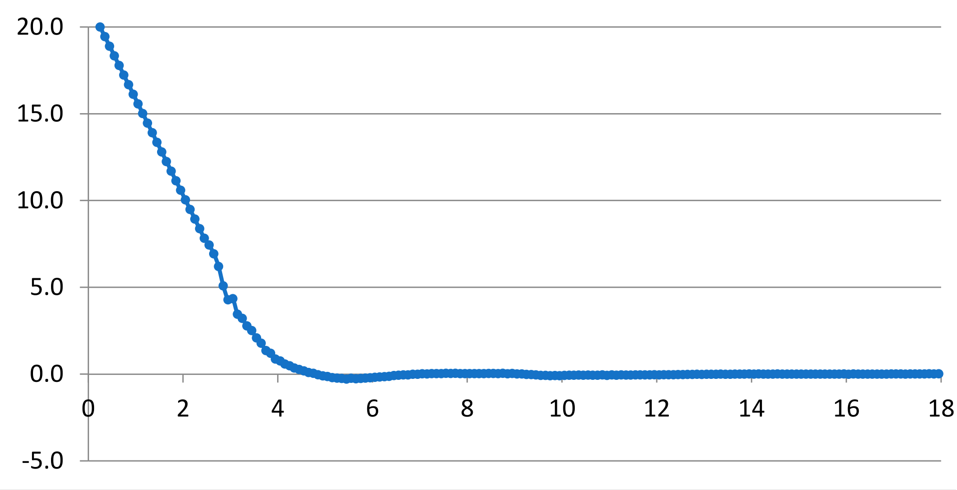

<table>
<tr><th>Interaction</th><th>Potential (energy [kcal·mol^{-1}] versus distance [Å])</th></tr>
<tr><td>E···B</td><td>
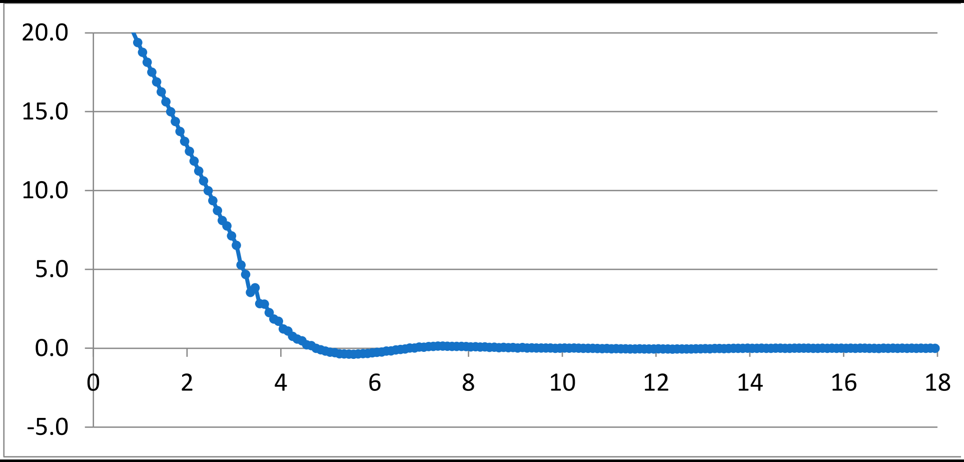

</td></tr>
<tr><td>E···Ad</td><td>
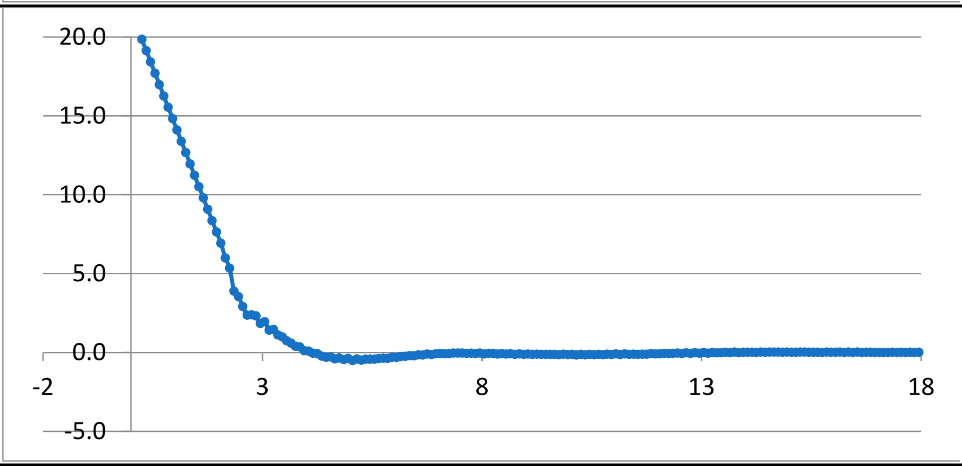

</td></tr>
<tr><td>E···Ae</td><td>
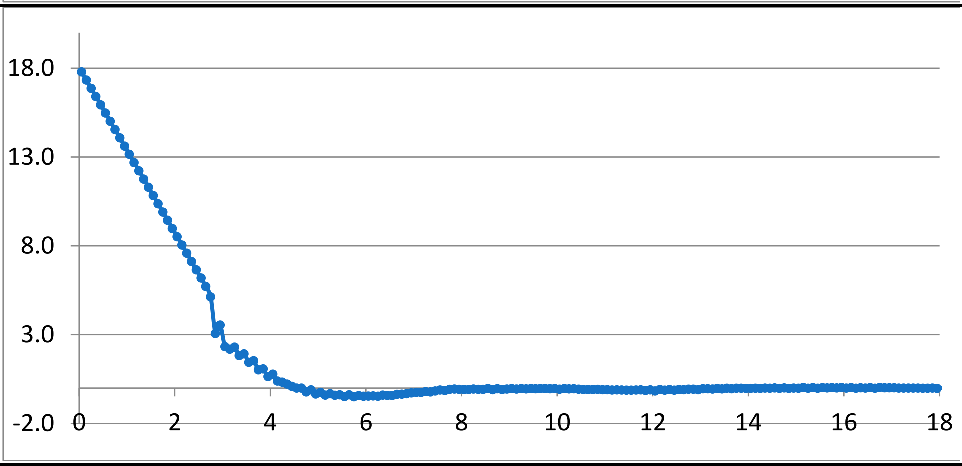

</td></tr>
</table>

Interaction	Potential (energy [kcal·mol^{-1}] versus distance [Å])
E···Ay	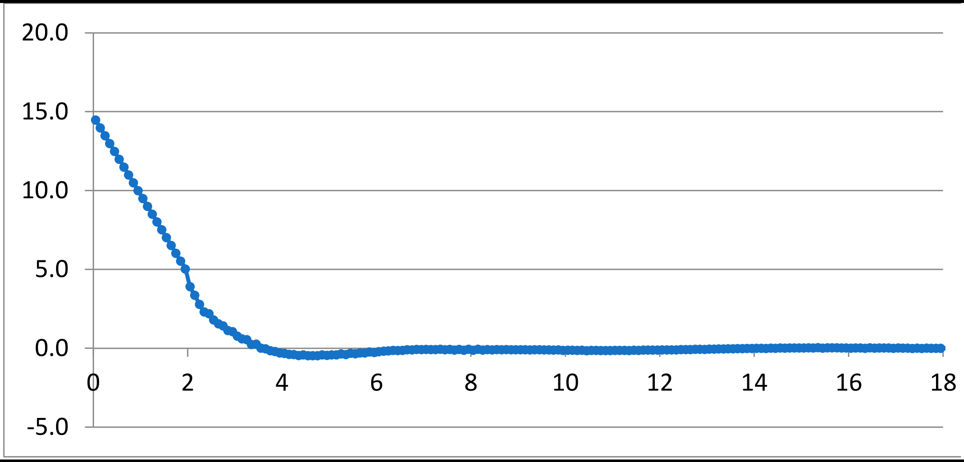
E···Y	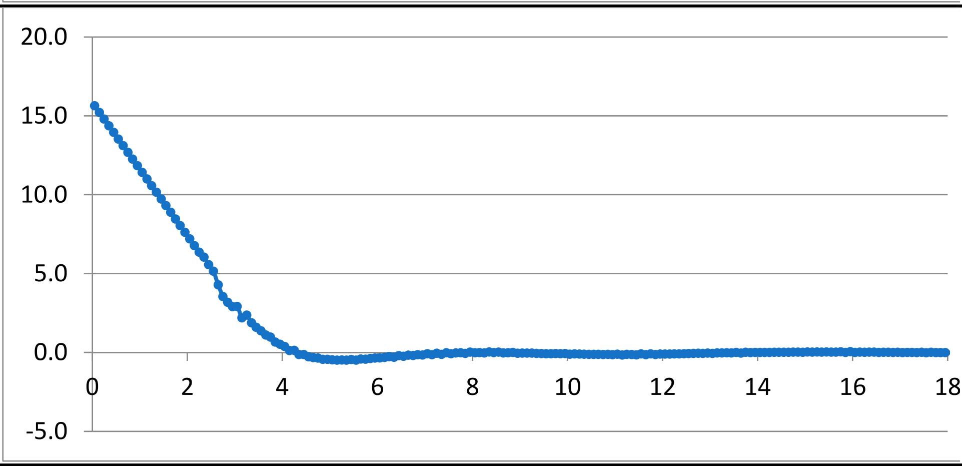
G···G	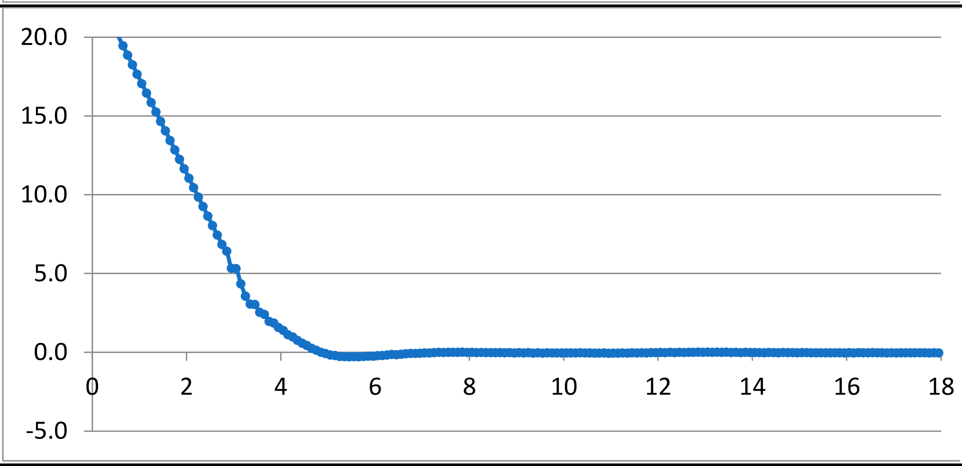

Interaction	Potential (energy [kcal·mol^{-1}] versus distance [Å])
G···B	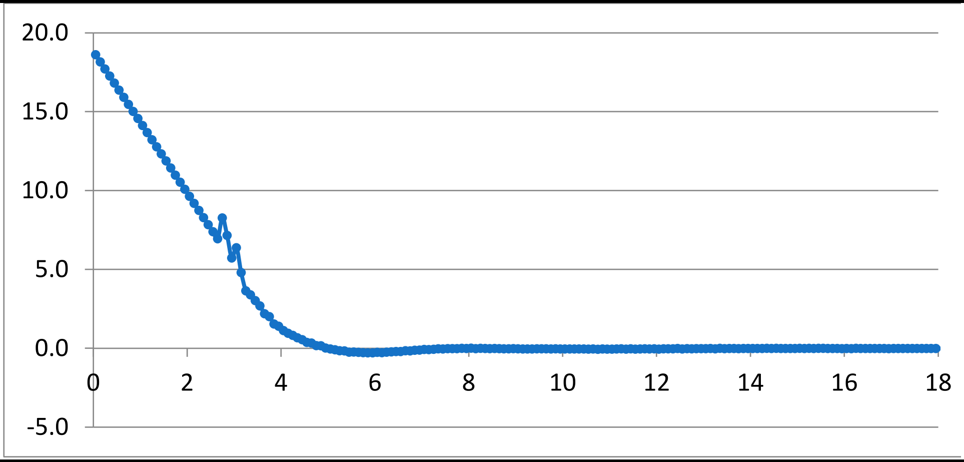
G···Ad	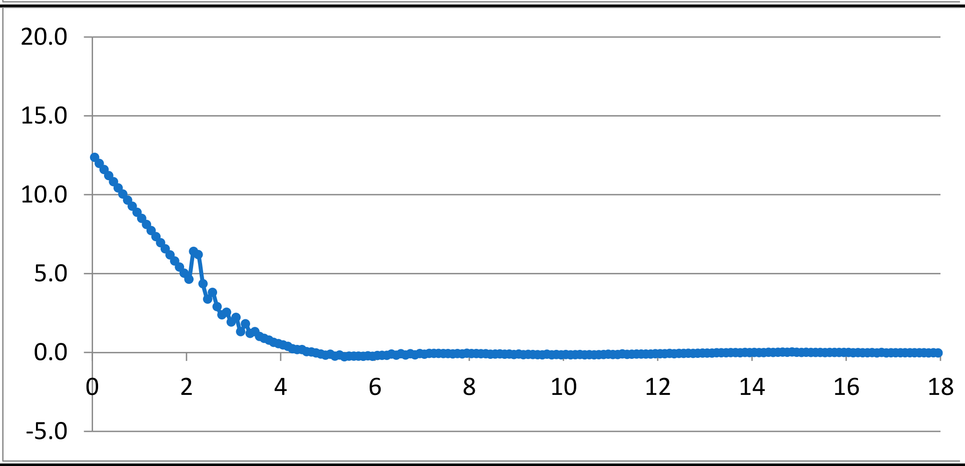
G···Ae	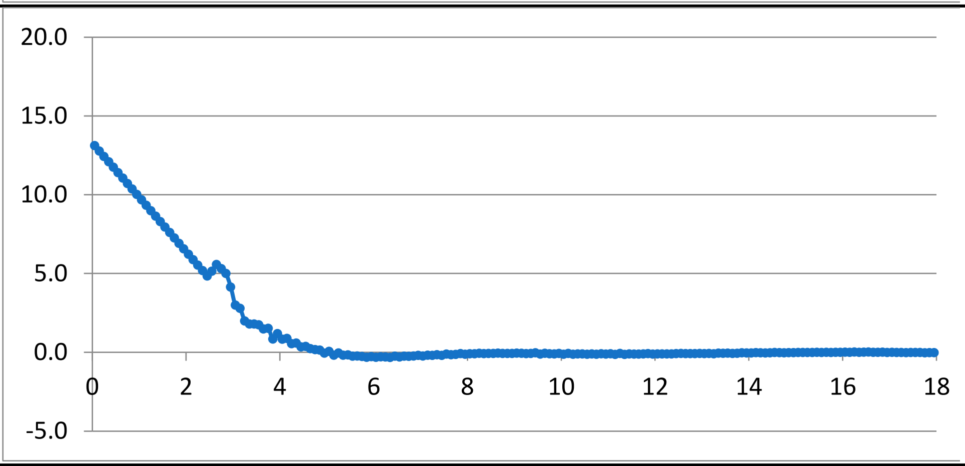

<table>
<tr><th>Interaction</th><th>Potential (energy [kcal·mol^{-1}] versus distance [Å])</th></tr>
<tr><td>G···Ay</td><td>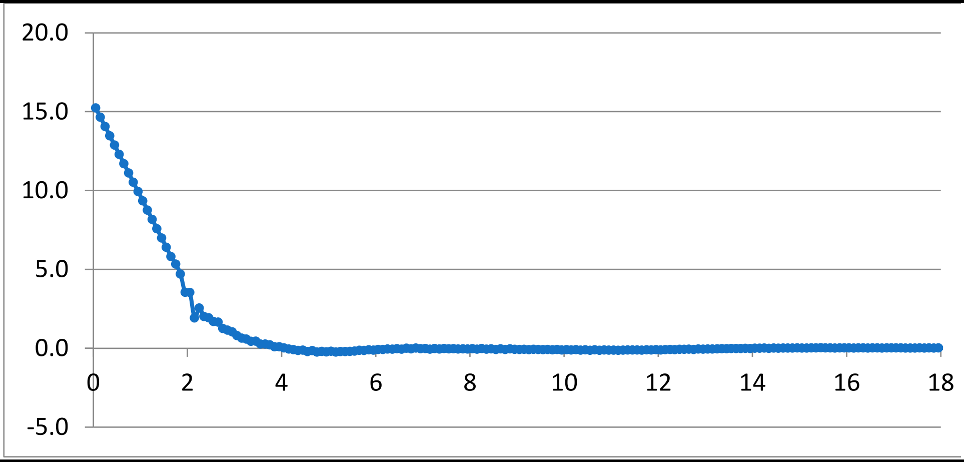
</td></tr>
<tr><td>G···Y</td><td>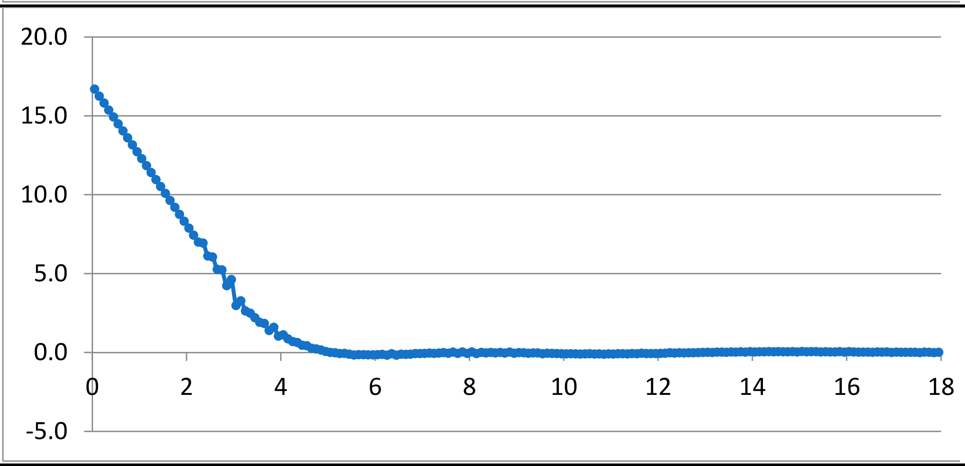
</td></tr>
<tr><td>B···B</td><td>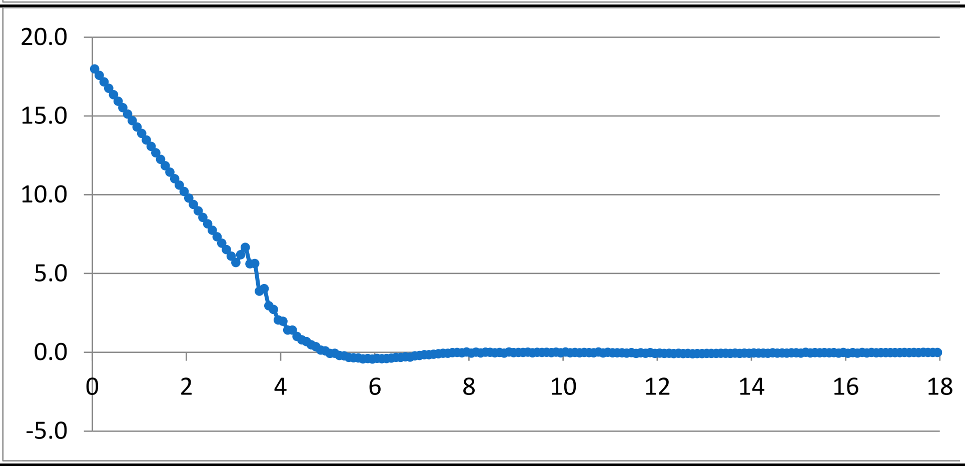
</td></tr>
</table>

<table>
<tr><th>Interaction</th><th>Potential (energy [kcal·mol^{-1}] versus distance [Å])</th></tr>
<tr><td>B···Ad</td><td>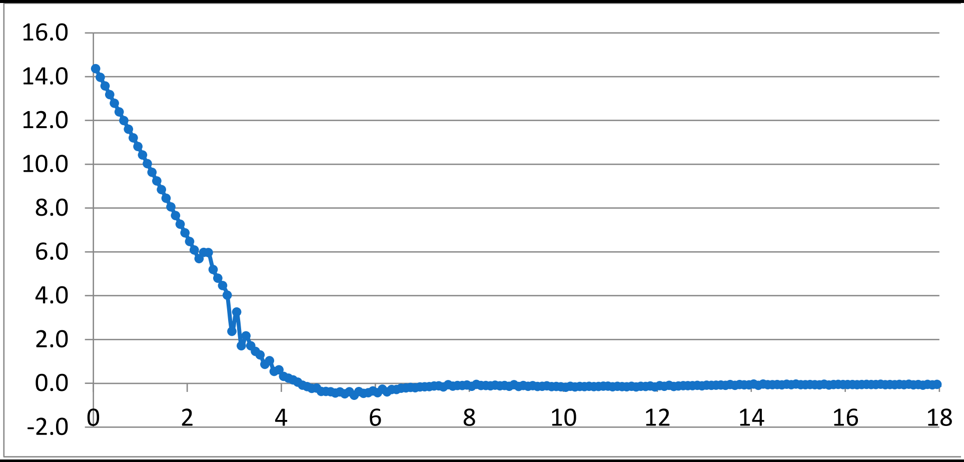
</td></tr>
<tr><td>B···Ae</td><td>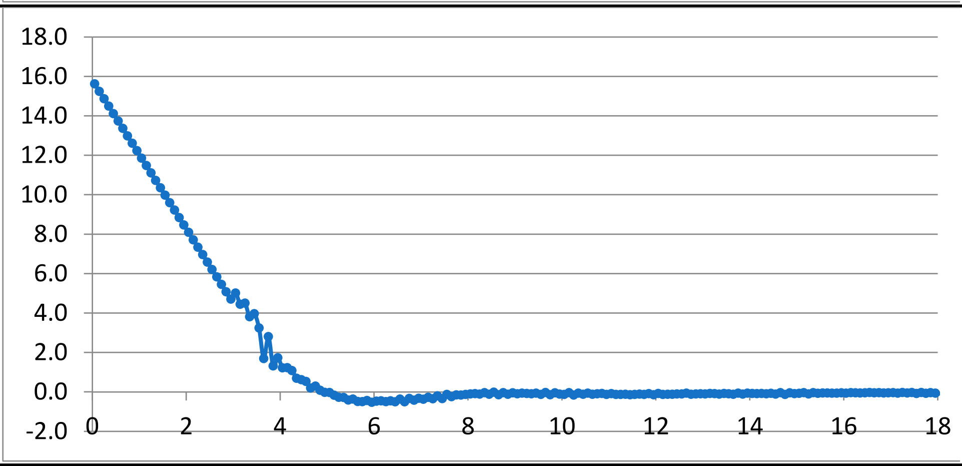
</td></tr>
<tr><td>B···Ay</td><td>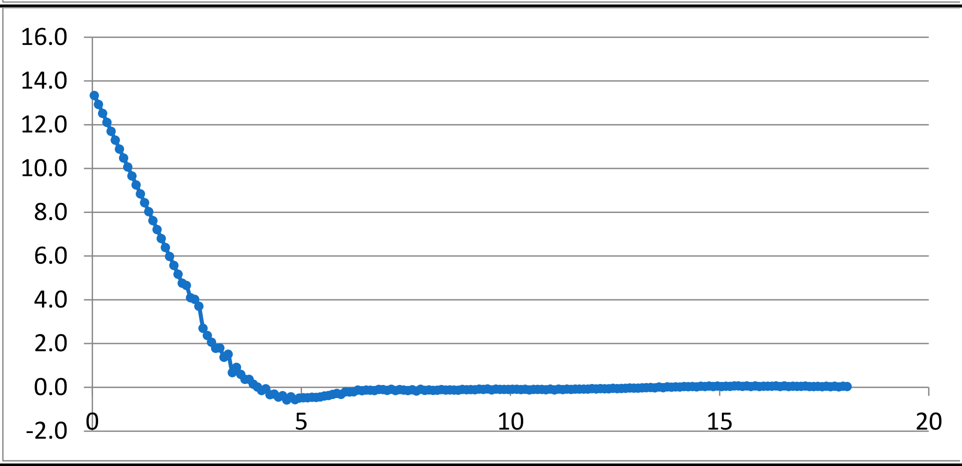
</td></tr>
</table>

<table>
<tr><th>Interaction</th><th>Potential (energy [kcal·mol^{-1}] versus distance [Å])</th></tr>
<tr><td>B···Y</td><td>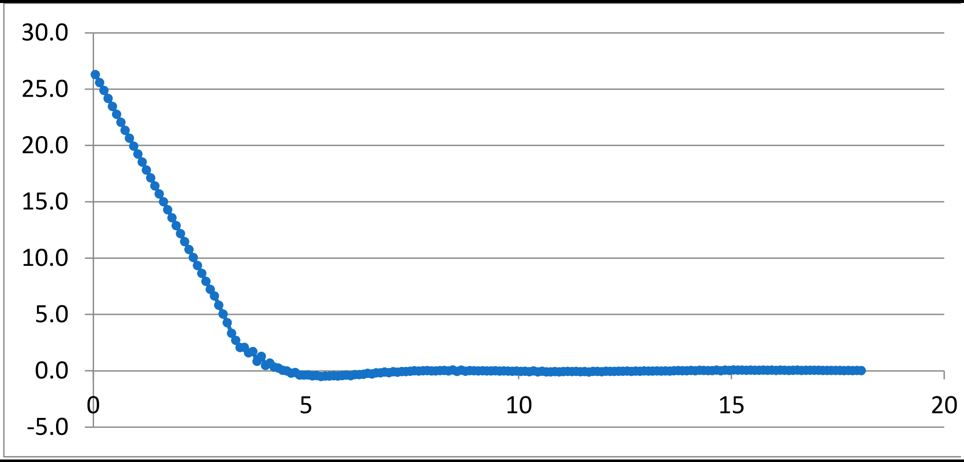
</td></tr>
<tr><td>Ad···Ad</td><td>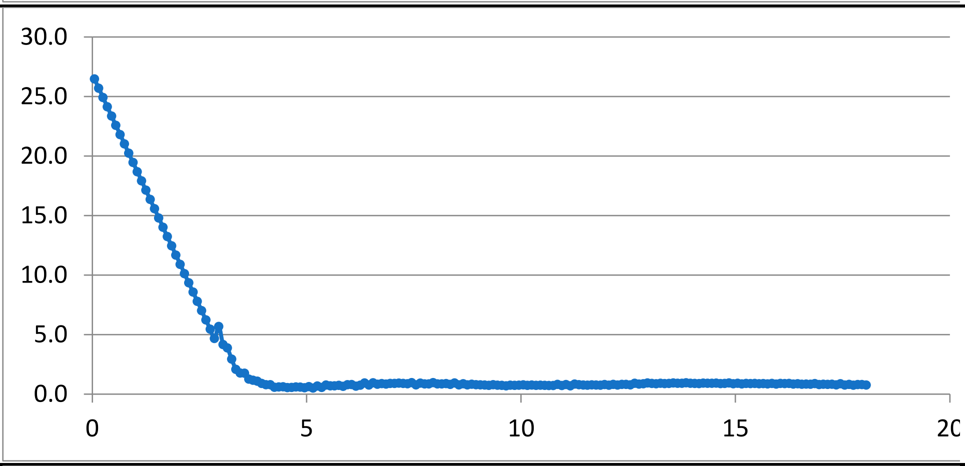
</td></tr>
<tr><td>Ad···Ae</td><td>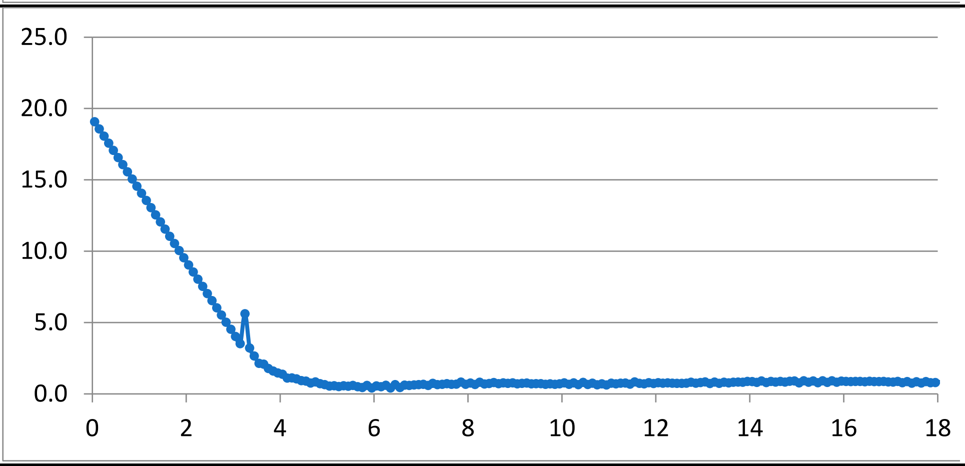
</td></tr>
</table>

Interaction	Potential (energy [kcal·mol^{-1}] versus distance [Å])
Ad···Ay	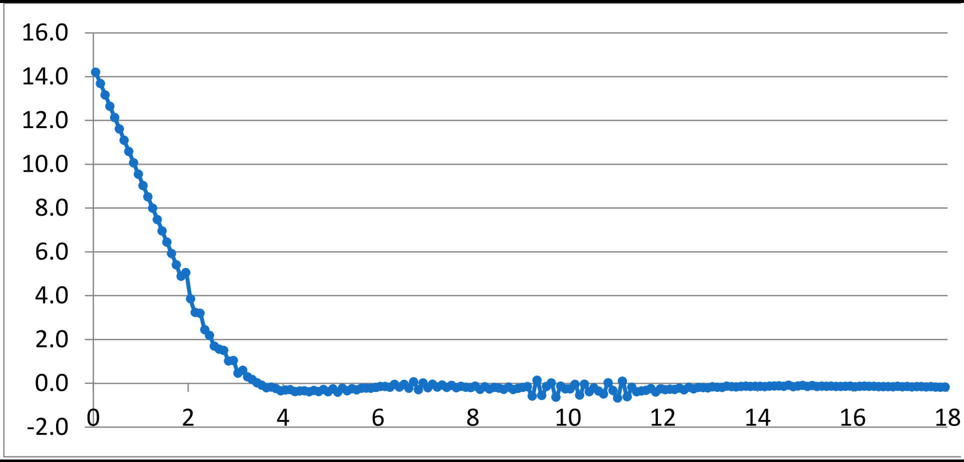
Ad···Y	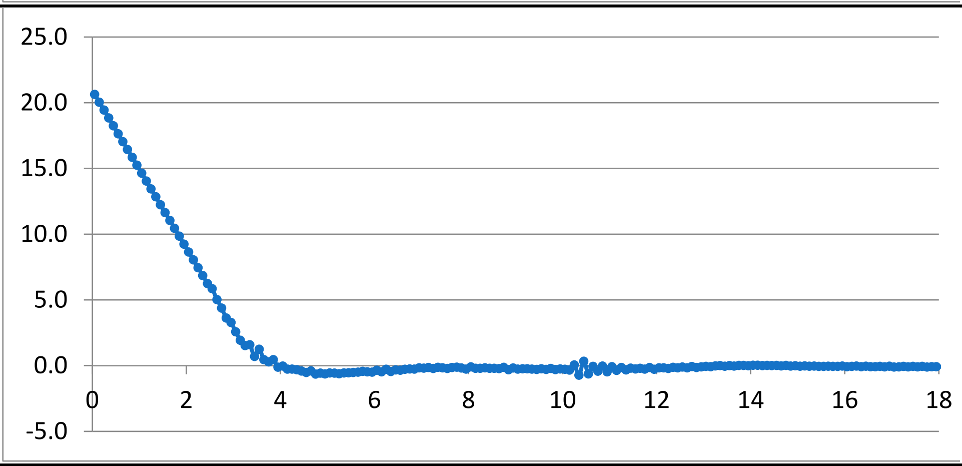
Ae···Ae	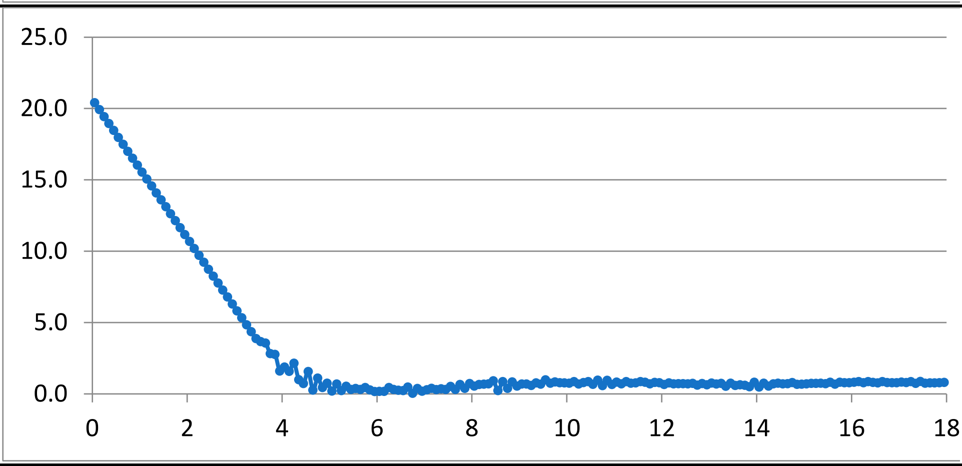

Interaction	Potential (energy [kcal·mol^{-1}] versus distance [Å])
Ae···Ay	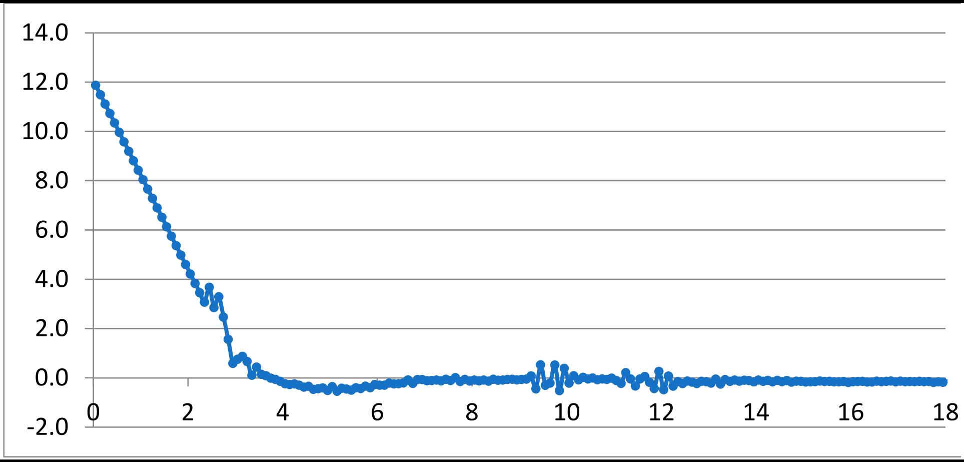
Ae···Y	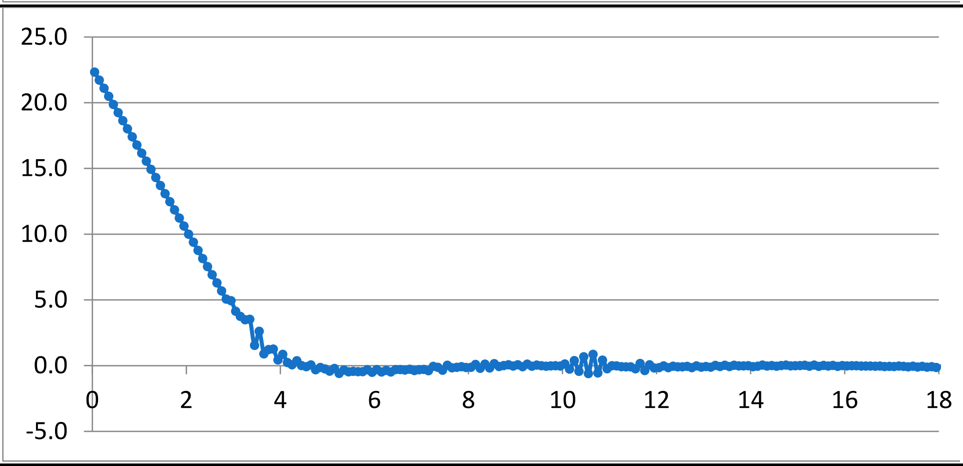
Ay···Ay	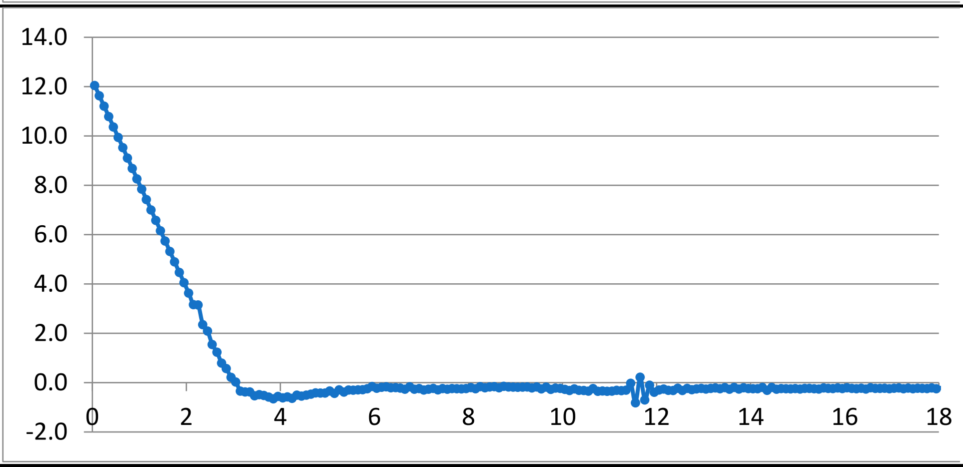

Interaction	Potential (energy [kcal·mol^{-1}] versus distance [Å])
Ay···Y	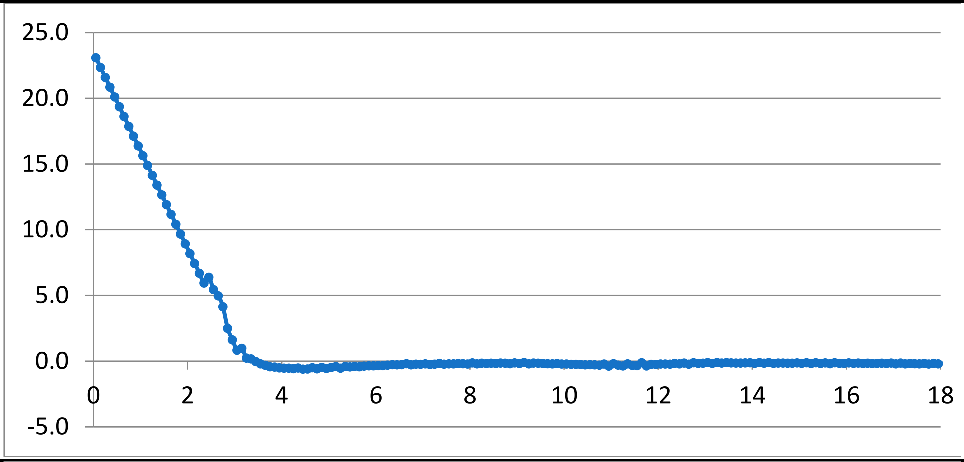
Y···Y	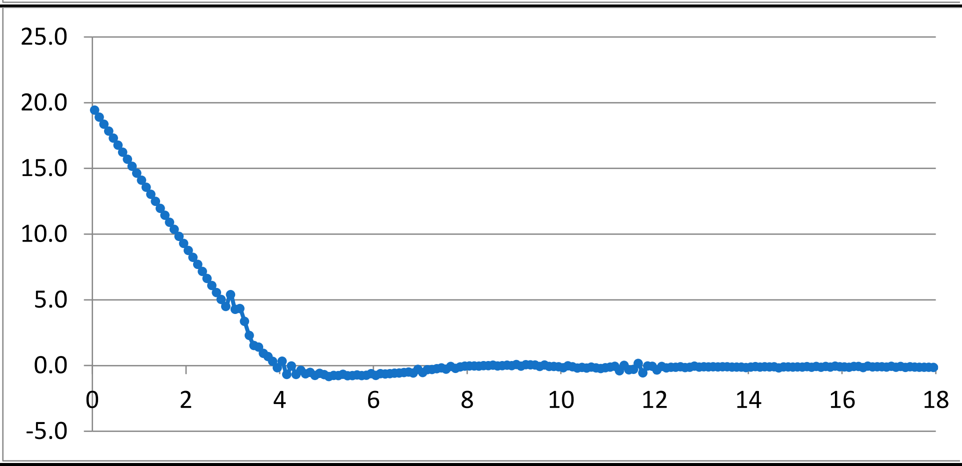

iv. Stability of developed EPO-CG force field

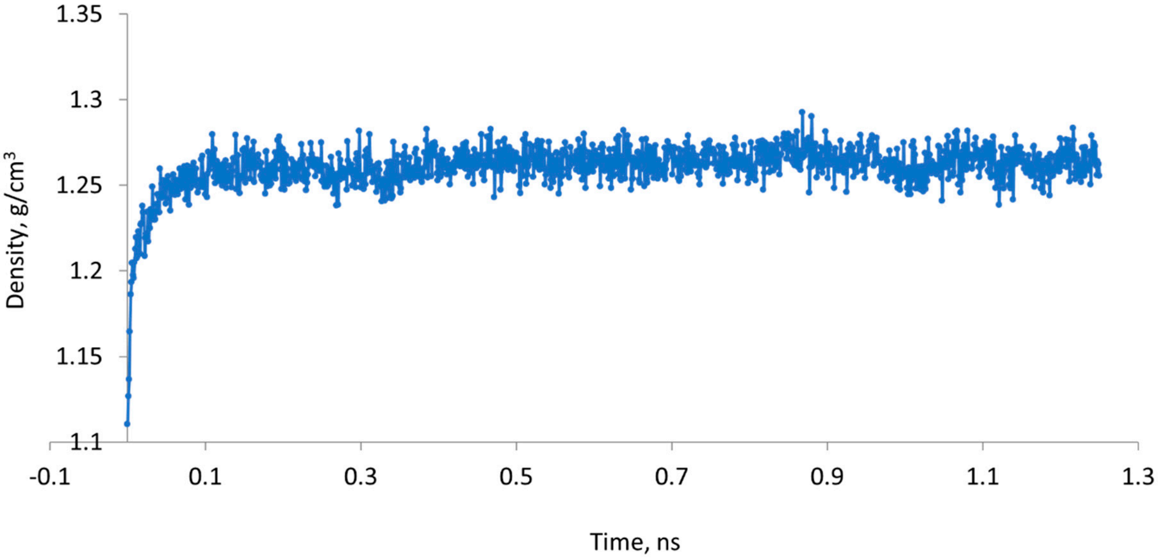

Figure S6. Density versus time in cross-linked CG epoxy system.

Supplementary Note S4: Epoxy graphene composites CG force field (EPO-GRP-CGff)

i. Non-bonded potentials

Non-bonded			
E ··· Gr	B ··· Gr	Ae ··· Gr	Y ··· Gr
G ··· Gr	Ad ··· Gr	Ay ··· Gr	

Table S5. List of potentials in the developed EPO-GRP-CGff forcefield for non-bonded interactions.

Interaction	Potential (energy [kcal·mol^{-1}] versus distance [Å])
E···Gr	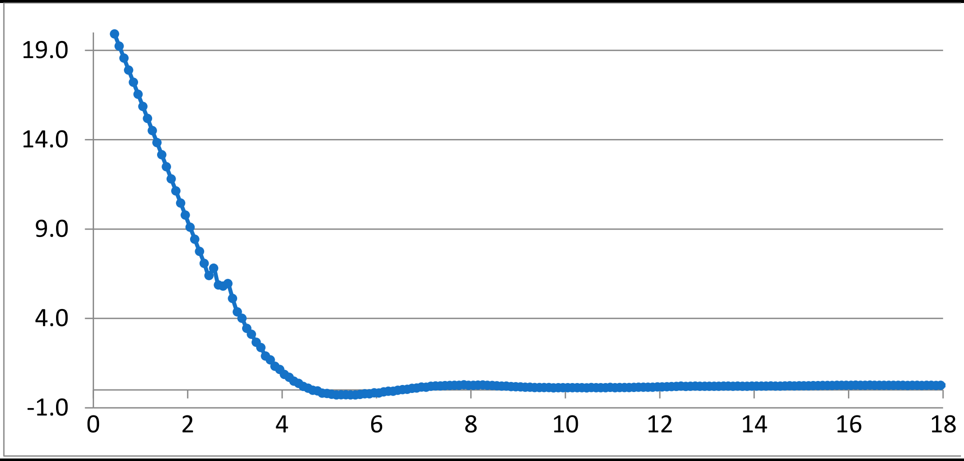
G···Gr	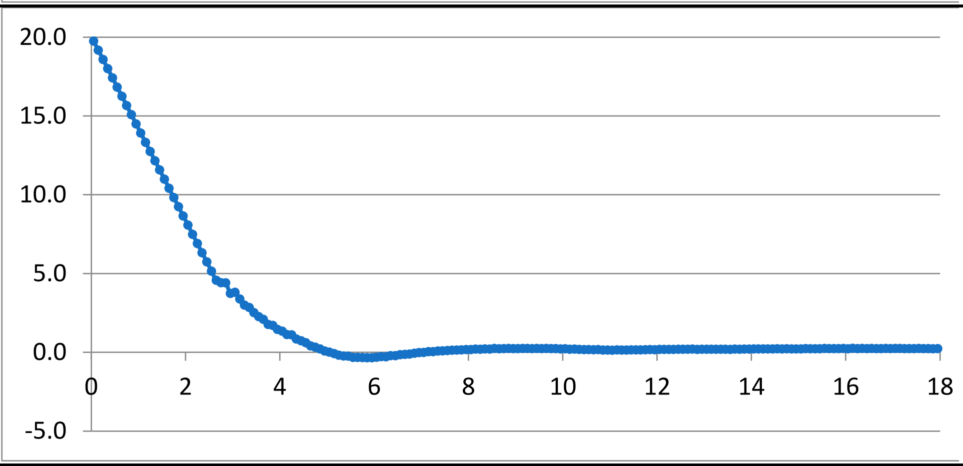
B···Gr	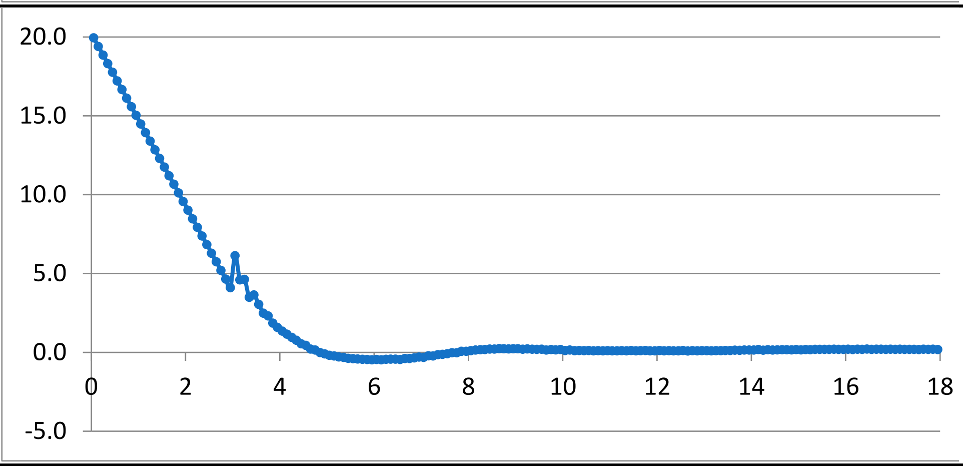

Interaction	Potential (energy [kcal·mol^{-1}] versus distance [Å])
Ad···Gr	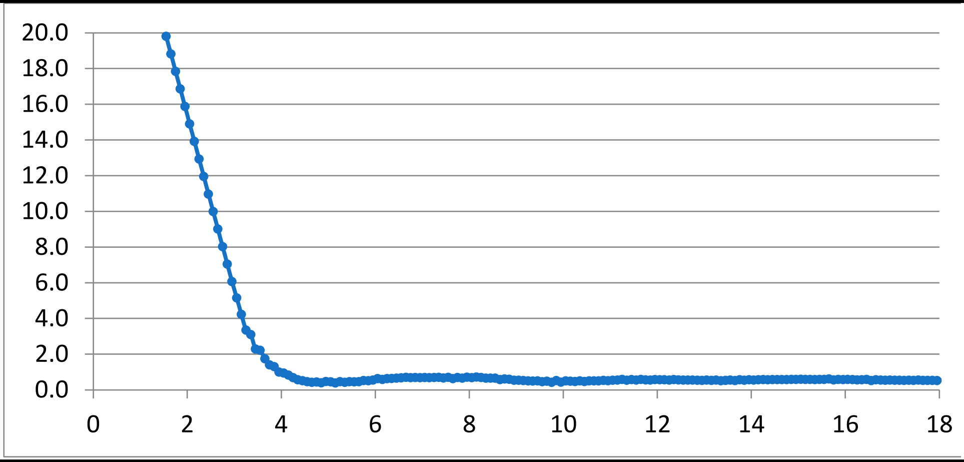
Ae···Gr	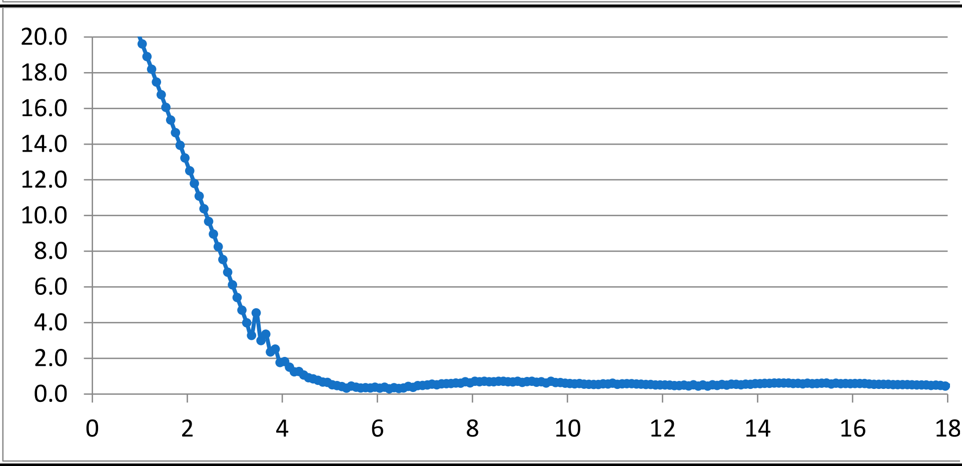
Ay···Gr	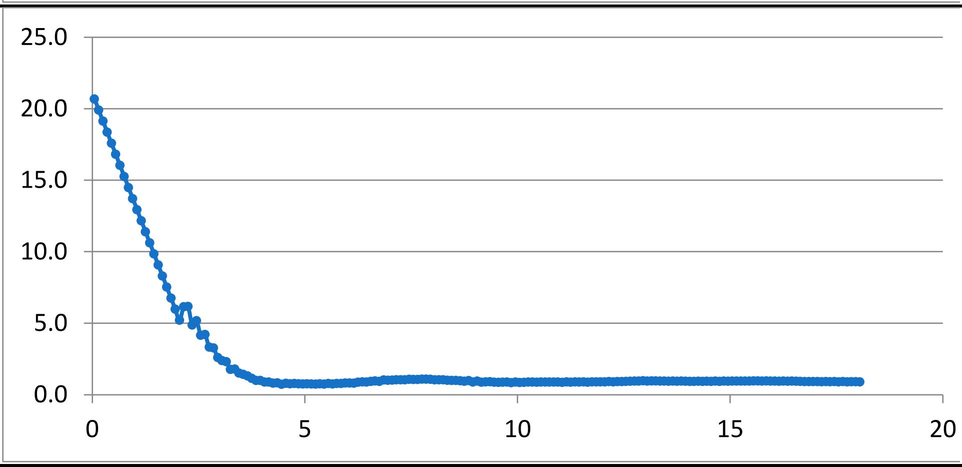

Interaction	Potential (energy [kcal·mol^{-1}] versus distance [Å])
Y···Gr	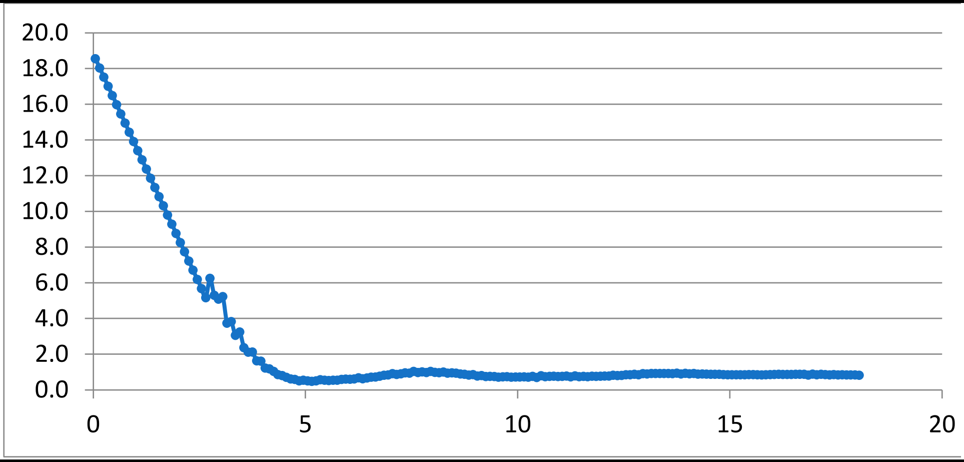

Supplementary Note S5: Tabulated results

Properties	AA MD	CG MD	Literature Ref
**Density (g/cm^3)**	1.155 (0.003)	1.24 (0.005)	1.07-1.20 [1]–[3]
**Glass transition temperature (K)**	387.87	390.40	380-390 [4]
**Young's modulus (GPa)**	2.999 (0.010)	1.419 (0.043)	2.5-5 [1]–[3], [5]–[8]
**Poisson's ratio (-)**	0.293 (0.010)	0.335 (0.012)	0.22-0.33 [2], [5], [7]
**Thermal conductivity (W/m-K)**	0.163 (0.005)	0.099 (0.003)	0.18-0.2 [9], [10]
**Specific heat capacity (kJ/kg-K)**	3.5	0.55	1.0-2.0 [11], [12]

Table S6. Comparison of thermophysical properties of pure epoxy resin obtained from all-atomistic, coarse-grained molecular dynamics and literature reference values.

Properties	CG MD	AA MD Ref	Experimental Ref
**Young' modulus (GPa)**	981-1009	500-1200 [13]	1000 [14], [15]
**Poisson's ratio (-)**	0.14-0.16	0.14-0.39 [13]	0.19 [16]
**Thermal conductivity (W/m-K)**	520-546	800-1000 [17]	2000-5000 [18], [19]
**Specific heat capacity (kJ/kg-K)**	0.526	–	0.700 [20]

Table S7. Comparison of thermophysical properties of monolayer graphene sheet obtained from coarse-grained molecular dynamics and literature experimental values.

wt.%	CG MD E (GPa)	Continuum (MF) E (GPa)	Continuum (FE) E (GPa)
**0.0**	1.419 (0.043)	1.419	1.419 (0.043)
**0.5**	1.452 (0.046)	1.442	1.436 (0.004)
**0.8**	1.462 (0.018)	1.461	1.453 (0.011)
**1.0**	1.466 (0.025)	1.464	1.459 (0.008)
**1.5**	1.496 (0.027)	1.497	1.490 (0.000)
**2.0**	1.549 (0.036)	1.530	1.519 (0.022)

Table S8. Comparison of Young's modulus obtained from coarse-grained and continuum simulations.

wt.%	CG MD<br>Poisson's ratio (GPa)	Continuum (MF)<br>E (GPa)	Continuum (FE)<br>E (GPa)
**0.0**	0.335 (0.012)	0.335	0.335 (0.012)
**0.5**	0.321 (0.006)	0.333	0.333 (0.000)
**0.8**	0.319 (0.005)	0.332	0.332 (0.000)
**1.0**	0.318 (0.006)	0.332	0.332 (0.000)
**1.5**	0.312 (0.004)	0.330	0.331 (0.000)
**2.0**	0.306 (0.014)	0.329	0.330 (0.001)

Table S9. Comparison of Poisson's ratio obtained from coarse-grained and continuum simulations.

wt.%	CG MD<br>λ (W/mK)	Continuum (MF)<br>λ (W/mK)	Continuum (FE)<br>λ (W/mK)
**0.0**	0.099 (0.003)	0.099	0.099 (0.003)
**0.5**	0.104 (0.003)	0.108	0.107 (0.000)
**0.8**	0.114 (0.002)	0.111	0.109 (0.001)
**1.0**	0.116 (0.001)	0.113	0.111 (0.001)
**1.5**	0.118 (0.001)	0.118	0.115 (0.000)
**2.0**	0.120 (0.002)	0.123	0.120 (0.001)

Table S10. Comparison of thermal conductivity obtained from coarse-grained and continuum simulations.

References

[1] C. Liu, W. Ning, L. Tam, and Z. Yu, "Understanding fracture behavior of epoxy-based polymer using molecular dynamics simulation," *J Mol Graph Model*, vol. 101, p. 107757, Dec. 2020, doi: 10.1016/j.jmgm.2020.107757.

[2] R. Feng and R. J. Farris, "The characterization of thermal and elastic constants for an epoxy photoresist SU8 coating," *J Mater Sci*, vol. 37, no. 22, pp. 4793–4799, 2002, doi: 10.1023/A:1020862129948.

[3] H. Yu, O. Balogun, B. Li, T. W. Murray, and X. Zhang, "Building embedded microchannels using a single layered SU-8, and determining Young's modulus using a laser acoustic technique," *Journal of Micromechanics and Microengineering*, vol. 14, no. 11, pp. 1576–1584, Nov. 2004, doi: 10.1088/0960-1317/14/11/020.

[4] A. Gavrielides, T. Duguet, M. Aufray, and C. Lacaze-Dufaure, "Model of the DGEBA-EDA Epoxy Polymer: Experiments and Simulation Using Classical Molecular Dynamics," *Int J Polym Sci*, vol. 2019, pp. 1–9, Feb. 2019, doi: 10.1155/2019/9604714.

[5] A. T. Al-Halhouli, I. Kampen, T. Krah, and S. Büttgenbach, "Nanoindentation testing of SU-8 photoresist mechanical properties," *Microelectron Eng*, vol. 85, no. 5–6, pp. 942–944, May 2008, doi: 10.1016/j.mee.2008.01.033.

[6] H. Lorenz, M. Laudon, and P. Renaud, "Mechanical characterization of a new high-aspect-ratio near UV-photoresist," *Microelectron Eng*, vol. 41–42, pp. 371–374, Mar. 1998, doi: 10.1016/S0167-9317(98)00086-0.

[7] F. E. H. Tay, J. A. van Kan, F. Watt, and W. O. Choong, “A novel micro-machining method for the fabrication of thick-film SU-8 embedded micro-channels,” *Journal of Micromechanics and Microengineering*, vol. 11, no. 1, pp. 27–32, Jan. 2001, doi: 10.1088/0960-1317/11/1/305.

[8] J. Hammacher, A. Fuelle, J. Flaemig, J. Saupe, B. Loechel, and J. Grimm, “Stress engineering and mechanical properties of SU-8-layers for mechanical applications,” *Microsystem Technologies*, vol. 14, no. 9–11, pp. 1515–1523, Oct. 2008, doi: 10.1007/s00542-007-0534-7.

[9] Z. Wang *et al.*, “Dielectric properties and thermal conductivity of epoxy composites using core/shell structured Si/SiO2/Polydopamine,” *Compos B Eng*, vol. 140, pp. 83–90, May 2018, doi: 10.1016/j.compositesb.2017.12.004.

[10] J. Ren *et al.*, “Enhanced thermal conductivity of epoxy composites by introducing graphene@boron nitride nanosheets hybrid nanoparticles,” *Mater Des*, vol. 191, p. 108663, Jun. 2020, doi: 10.1016/j.matdes.2020.108663.

[11] A. C. Ackermann, M. Fischer, A. Wick, S. Carosella, B. L. Fox, and P. Middendorf, “Mechanical, Thermal and Electrical Properties of Epoxy Nanocomposites with Amine-Functionalized Reduced Graphene Oxide via Plasma Treatment,” *Journal of Composites Science*, vol. 6, no. 6, p. 153, May 2022, doi: 10.3390/jcs6060153.

[12] X. Wang, Q. Wang, L. Gao, and Y. Jia, “Effects of key thermophysical properties on the curing uniformity of carbon fiber reinforced resin composites,” *e-Polymers*, vol. 18, no. 1, pp. 19–26, Jan. 2018, doi: 10.1515/epoly-2017-0104.

[13] S. Thomas, K. M. Ajith, S. U. Lee, and M. C. Valsakumar, “Assessment of the mechanical properties of monolayer graphene using the energy and strain-fluctuation methods,” *RSC Adv*, vol. 8, no. 48, pp. 27283–27292, 2018, doi: 10.1039/C8RA02967A.

[14] C. Lee, X. Wei, J. W. Kysar, and J. Hone, “Measurement of the Elastic Properties and Intrinsic Strength of Monolayer Graphene,” *Science (1979)*, vol. 321, no. 5887, pp. 385–388, Jul. 2008, doi: 10.1126/science.1157996.

[15] Q. Cao *et al.*, “A Review of Current Development of Graphene Mechanics,” *Crystals (Basel)*, vol. 8, no. 9, p. 357, Sep. 2018, doi: 10.3390/cryst8090357.

[16] A. Politano and G. Chiarello, “Probing the Young’s modulus and Poisson’s ratio in graphene/metal interfaces and graphite: a comparative study,” *Nano Res*, vol. 8, no. 6, pp. 1847–1856, Jun. 2015, doi: 10.1007/s12274-014-0691-9.

[17] L. Wang and H. Sun, “Thermal conductivity of silicon and carbon hybrid monolayers: a molecular dynamics study,” *J Mol Model*, vol. 18, no. 11, pp. 4811–4818, Nov. 2012, doi: 10.1007/s00894-012-1482-4.

[18] A. A. Balandin, “Thermal properties of graphene and nanostructured carbon materials,” *Nat Mater*, vol. 10, no. 8, pp. 569–581, Aug. 2011, doi: 10.1038/nmat3064.

[19] S. Chen *et al.*, “Thermal conductivity of isotopically modified graphene,” *Nat Mater*, vol. 11, no. 3, pp. 203–207, Mar. 2012, doi: 10.1038/nmat3207.

[20] Q.-Y. Li *et al.*, “Measurement of specific heat and thermal conductivity of supported and suspended graphene by a comprehensive Raman optothermal method,” *Nanoscale*, vol. 9, no. 30, pp. 10784–10793, 2017, doi: 10.1039/C7NR01695F.
